# Adaptive immune responses against common viruses are sustained and functional in end-of-life patients

**DOI:** 10.1016/j.isci.2025.112082

**Published:** 2025-02-21

**Authors:** Anna Olofsson, Marion Humbert, Rokeya S. Rekha, Maria Helde Frankling, Fridtjof Lund-Johansen, Peter Bergman, Linda Björkhem-Bergman, Annika C. Karlsson

**Affiliations:** 1 Division of Clinical Microbiology, Department of Laboratory Medicine, Karolinska Institutet, Stockholm, Sweden; 2 Center for Infectious Medicine, Department of Medicine Huddinge, Karolinska Institutet, Stockholm, Sweden; 3 Division of Clinical Immunology, Department of Laboratory Medicine, Karolinska Institutet, Stockholm, Sweden; 4 Division of Clinical Geriatrics, Department of Neurobiology, Care Sciences and Society, Karolinska Institutet, Stockholm, Sweden; 5 Theme Cancer, Karolinska University Hospital, Stockholm, Sweden; 6 Institute of Clinical Medicine, University of Oslo, Department of Cancer Immunology, Institute for Cancer Research, Oslo University Hospital, Oslo, Norway; 7 Department of Immunology, Oslo University Hospital, ImmunoLingo Convergence Center, Institute of Clinical Medicine, University of Oslo, Oslo, Norway; 8 Department of Clinical Immunology and Transfusion Medicine, Karolinska University Hospital, Stockholm, Sweden; 9 Research and Development Unit/Palliative Care, Stockholms Sjukhem, Mariebergsgatan 22, Stockholm, Sweden

**Keywords:** Health sciences, Medicine, Medical specialty, Immunology

## Abstract

Viral infections occur with increased frequency in patients in palliative care, impacting their quality of life and increasing mortality rates. Still, the function of the immune system has never been thoroughly studied at the end of life. We investigated virus-specific humoral and cellular immune responses in elderly end-of-life patients (*n* = 38) and controls (*n* = 28). Virus-specific T cell responses were characterized using high-parameter flow cytometry, after stimulation with cytomegalovirus (CMV) and human coronavirus OC43 peptides. Although some virus-specific T cells from patients exhibited elevated expression of costimulatory and coinhibitory molecules, their functional profile remained largely intact compared to controls. The expression of the cytotoxic markers Granzyme B, CD107a, and 2B4 on CMV-specific T cells correlated closely with survival time. Significantly, our data demonstrate that both humoral and cellular immunity remain responsive and functional against common viruses in end-of-life patients.

## Highlights

• Humoral immunity against acute and chronic viruses is sustained at the end of life
• Memory T cells display elevated activation but remain functional at the end of life
• CMV-specific CD8^+^ T cells are more polyfunctional in end-of-life patients
• The cytotoxic capacity of CMV-specific T cells is linked to patients’ survival time

## Graphical abstract

**Figure:**
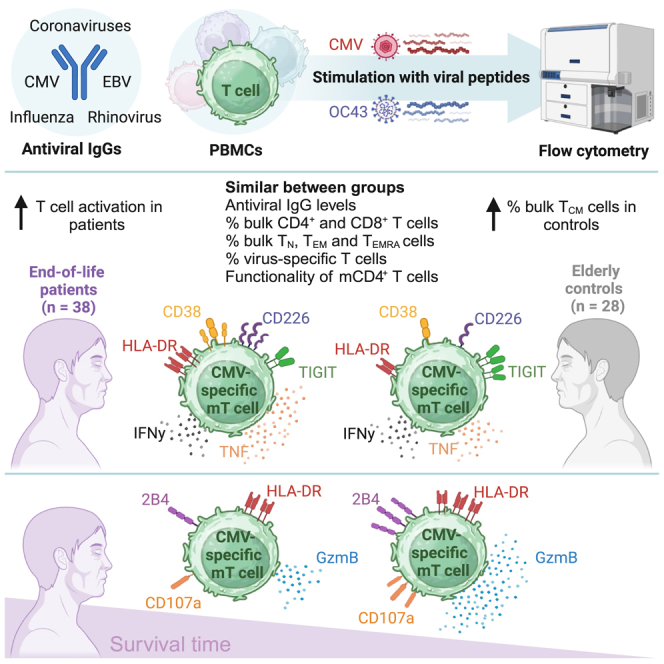

## Introduction

It is widely recognized that several essential bodily functions deteriorate in end-of-life patients, including cognition, gastrointestinal health, and renal function.^1^ Infections in end-of-life patients are common and lead to reduced quality of life and increased mortality.^2^^,^^3^^,^^4^ The decline of the immune system can occur at various levels.^5^^,^^6^^,^^7^^,^^8^ For instance, cancer drugs may harm mucosal surfaces, potentially leading to invasive bacterial infections such as sepsis, pyelonephritis, or pneumonia.^9^ Additionally, cachexia, a syndrome causing muscle loss, is a prevalent condition in end-of-life patients, which can result in the breakdown of essential immune proteins, including antibodies and complement factors, thereby increasing susceptibility to infections.^10^ Whether aging of the immune system is further aggravated in the final days and weeks in elderly patients receiving palliative care is unknown.

Age is an independent risk factor for severe infections the last year in life in older adults^11^ and is generally associated with the severity of seasonal viral respiratory tract infections.^12^^,^^13^^,^^14^ This includes influenza, rhinoviruses, and the recent pandemic virus SARS-CoV-2, responsible for COVID-19. During the early phase of the pandemic, COVID-19 spread rapidly in nursing homes for elderly, causing significant morbidity and mortality among this vulnerable group.^15^^,^^16^ Notably, advanced age was the strongest risk factor for severe COVID-19 infection, likely due to a deteriorating immune system and a high frequency of concomitant diseases.^17^^,^^18^ Another infectious manifestation in the elderly and frail population is the reactivation of latent viruses from the human *Herpesviridae* family,^19^^,^^20^ including cytomegalovirus (CMV), Epstein-Barr virus (EBV), and varicella-zoster virus (VZV). These reactivations can lead to severe symptoms such as fever, pain, and, in severe cases, encephalitis, retinitis, and death.^21^^,^^22^^,^^23^ The protection against viral infections relies on intricate combinations of innate and adaptive immune pathways.^20^^,^^24^^,^^25^^,^^26^ The adaptive immune response, including neutralizing viral-specific antibodies produced by B cells and memory (m)T cells, are orchestrated by professional antigen-presenting cells, like dendritic cells, and T helper cells. Importantly, there is limited information on whether T-cell-mediated immunity composed by mCD4^+^ T helper cells and mCD8^+^ T cells, known for their capacity to kill virally infected cells, is preserved during aging, especially in end-of-life patients.

We have recently demonstrated that the quality of the adaptive mCD4^+^ T cell responses against seasonal human coronaviruses (HCoV) OC43 and SARS-CoV-2 peaked at 6 years and declined with age, in healthy subjects.^27^ Building on this knowledge, we hypothesized that the T cell response to common viral antigens would deteriorate in end-of-life patients, compared with elderly controls. Our hypothesis aligns with our extensive clinical observations in patients receiving palliative care, where reactivation of latent viral infections becomes more frequent as death approaches.^11^^,^^28^ Thus, we postulated that the immune system also deteriorates during this critical period, potentially compromising the defense against viruses in the final weeks and days of life. To test this hypothesis, we performed a comprehensive immunological phenotyping of the mCD4^+^ and mCD8^+^ T cell responses to HCoV-OC43 and CMV in end-of-life patients admitted to an in-patient palliative care ward, compared with elderly healthy controls. Adaptive humoral immunity directed against HCoV-OC43, CMV, and other common viruses was also assessed in these two groups. We focused on HCoV-OC43 and CMV due to their high prevalence in the Swedish population, distinct pathogeneses (acute vs. lifelong latent infection), and potential to cause severe disease in older adults and the immunocompromised.^20^^,^^29^^,^^30^ Our study primarily aimed to characterize cellular immunity mediated by mT cell subsets using high-parameter flow cytometry. This approach allowed us to simultaneously assess the phenotype and functional capacity of antigen-specific mCD4^+^ and mCD8^+^ T cells at the end of life.

## Results

### Cohort description

**Figure 1:**
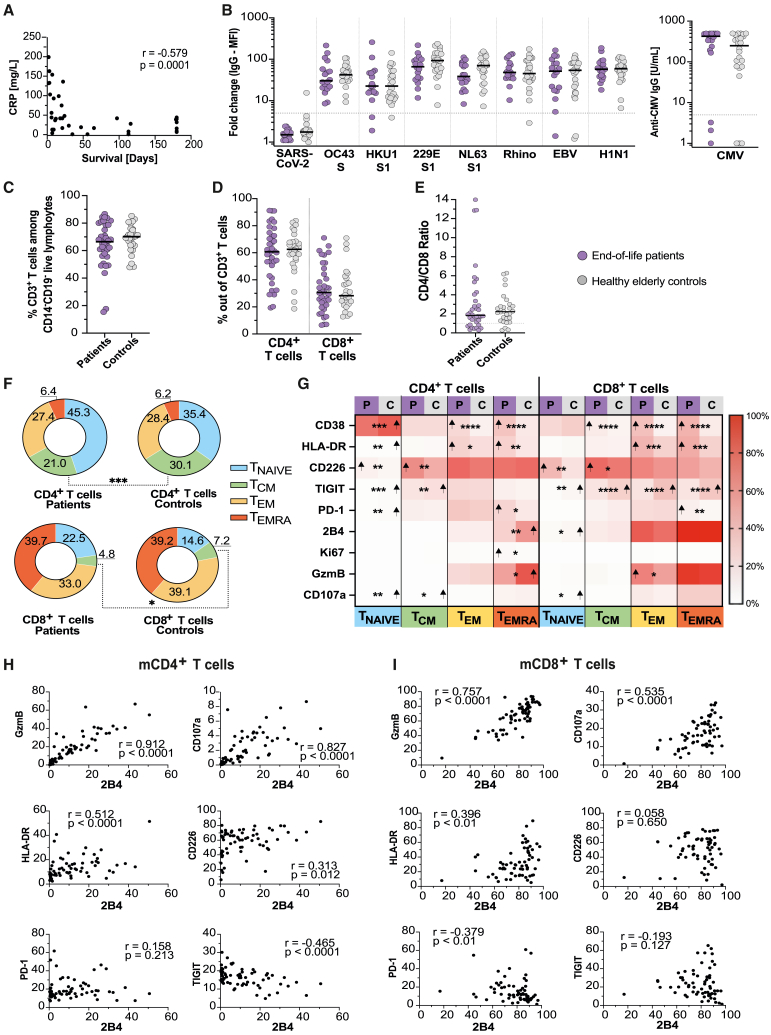
Fewer central memory T cells and increased activation in memory T cells at the end of life (A) Spearman correlation between CRP plasma concentration and survival time in the end-of-life patient cohort (*n* = 38). (B) Antibody levels (total IgG) against common viruses in end-of-life patients (*n* = 18) and elderly controls (*n* = 24). Results shown as either fold change of median fluorescence intensity (MFI) relative to background (left) or as U/mL (anti-CMV, right). Gray dotted line represents the threshold for a positive response. Mann Whitney U test. (C) Frequency of CD3^+^ T cells among live CD14^−^CD19^−^lymphocytes in end-of-life patients (*n* = 36) and elderly controls (*n* = 28). Mann Whitney U test. (D) Frequency of CD4^+^ T cells and CD8^+^ T cells among live CD14^−^CD19^−^CD3^+^ T cells in end-of-life patients (*n* = 36) and elderly controls (*n* = 28). Mann Whitney U test. (E) Ratio of CD4^+^ and CD8^+^ T cells for patients (*n* = 36) and controls (*n* = 28). Ratio was calculated using the frequency of CD4^+^ and CD8^+^ T cells acquired from flow cytometry. Mann Whitney U test. (F) Donut graph showing the mean frequency of naive and memory subsets among bulk CD4^+^ or CD8^+^ T cells in end-of-life patients (*n* = 36) and elderly controls (*n* = 28). TNAIVE, naive T cells; TCM, central memory; TEM, effector memory; TEMRA, effector memory cells re-expressing CD45RA. Mann Whitney U test for each population. Data are represented as mean frequency. (G) Heatmap showing the median frequency of unstimulated cells within the T cell subsets expressing activation, exhaustion, and functional markers. Shown for end-of-life patients (P; *n* = 36) and elderly controls (C; *n* = 28) with stars marking identified significant differences and arrows marking the population with higher median frequency. TNaive, naive T cells; TCM, central memory T cells; TEM, effector memory T cells; TEMRA, effector memory T cells re-expressing CD45RA. Mann Whitney U test. Data represented as median. (H) Spearman correlation of the frequency of 2B4 expression against the frequency of cytotoxic (top row), activation (middle row), and inhibitory (bottom row) markers on bulk mCD4+ T cells. Includes data from end-of-life patients (*n* = 36) and elderly controls (*n* = 28). (I) Spearman correlation of the frequency of 2B4 expression against the frequency of cytotoxic (top row), activation (middle row), and inhibitory (bottom row) markers on bulk mCD8+ T cells. Includes data from end-of-life patients (*n* = 36) and elderly controls (*n* = 28). Correlation graphs show spearman correlation coefficient (r) and *p* value (p). ∗*p* < 0.05; ∗∗*p* < 0.01; ∗∗∗*p* < 0.001; ∗∗∗∗*p* < 0.0001. The median (black line) is shown when applicable.

To characterize cellular immunity mediated by mT cell subsets in the end-of-life, we obtained samples from patients admitted to an in-patient palliative care ward (*n* = 38) and elderly healthy controls (*n* = 28), presented in Table 1 and Table S1. In brief, the prevailing diagnosis in the patient cohort was cancer, with 82% of patients diagnosed with some type of cancer. Non-cancer conditions consisted of chronic obstructive lung disease, heart failure, and amyotrophic lateral sclerosis (ALS). The comorbidities of importance in the patient group were kidney failure, diabetes, heart failure, and COPD (Table S1). Median age was significantly higher (9 years) in the patient cohort, compared to the control cohort, consisting of non-hospitalized, healthy elders living at home. However, the patient cohort had a greater age span, including individuals both younger and older than the controls, and when stratifying the patients by survival time, the age distribution was comparable between the two resulting patient groups. Patients had significantly higher levels of C-reactive protein (CRP) and lower levels of albumin and vitamin D (VitD) compared to the elderly controls. The median survival time after inclusion in the patient cohort was 21 days. CRP was inversely correlated, whereas albumin and VitD levels were positively correlated, with survival time in end-of-life patients (Figures 1A and S1A). Age was inversely correlated with CRP and positively correlated with albumin levels across both cohorts (Figure S1B). Samples were collected in 2017 and from January to October in 2020 (Table 1). Sampling was finalized early into the second wave of SARS-CoV-2 in Sweden and before the existence of an SARS-CoV-2 vaccine.^31^

**Table 1:** Clinical data for the end-of-life patients and healthy elderly controls

	Patients *n* = 38	Healthy controls *n* = 28	*p* value	≤30-day survival among patients *n* = 22	>30-day survival among patients *n* = 16	*p* value
Age in years (range)	77 (53–93)	66 (60–83)	<0.05	75 (53–93)	78 (53–90)	0.77
Females, n (%)	29 (76%)	16 (57%)	0.12	17 (77%)	12 (75%)	0.87
Available plasma, n (% of samples)	18 (47%)	24 (85%)	N/A	10 (45%)	8 (50%)	N/A
CMV seropositivity, n (% of available plasma)	15 (83%)	21 (88%)	0.70	9 (90%)	6 (75%)	0.40
Samples collected in 2017, n (%)^a^	20 (53%)	4 (14%)	<0.01	12 (55%)	8 (50%)	0.78
CRP, mg/L (IQR)	40 (13–80)	1 (1–2)	<0.0001	52 (38–125)	16.5 (8–34)	<0.001
Albumin, g/L (IQR)	25 (19–29)	38 (36–40)	<0.0001	21 (19–24)	29 (26–31)	<0.01
25-OH-Vitamin D, nmol/L (IQR)	43 (28–60)	81 (69–92)	<0.0001	42 (29–52)	57 (32–76)	0.06
Survival time, days from inclusion (IQR)	21 (7–114)	N/A	N/A	9 (6–20)	148 (89–180)	<0.0001

**Disease**						

Non-cancer, n (%)	7 (18%)	N/A	N/A	2	5	N/A
Cancer, n (%)	31 (82%)	N/A	N/A	20	11	N/A

**Non-cancer conditions**						

COPD	4	N/A	N/A	2	2	N/A
Heart failure	2	N/A	N/A	N/A	2	N/A
ALS	1	N/A	N/A	N/A	1	N/A

**Types of cancer**						

Lung cancer	6	N/A	N/A	6	N/A	N/A
GI cancer	7	N/A	N/A	6	1	N/A
Hormonal dependent cancer (prostate, breast, gynecological)	8	N/A	N/A	5	3	N/A
Hematological cancer	2	N/A	N/A	N/A	2	N/A
Other	8	N/A	N/A	3	5	N/A

Values show median and interquartile range (IQR) or range or sample size (n) and percentage (%) within parenthesis. The patient cohort was further stratified by survival time (≤30 days or >30 days) post-sampling. Differences between patients and healthy controls, and between patients with ≤30-day survival and patients with >30-day survival, were assessed using χ2 test for categorical variables and t test for continuous variables.

a The remaining samples were collected in 2020 (last samples collected in October 2020). All samples collected in 2020 had plasma available for antibody screening. CRP, C-reactive protein; COPD, chronic obstructive lung disease; ALS, amyotrophic lateral sclerosis; GI, gastrointestinal; N/A, not applicable.

### Antibody titers remain intact at the end of life

To determine if the antiviral humoral immunity was affected in end-of-life patients compared to healthy elders, antibody levels against common viruses were measured in all donors with available plasma (43/66, 65%; Figure 1A). One positive and two borderline positive antibody responses against SARS-CoV-2 were observed among the control samples (Figure 1B). In the patient cohort, influenza A H1N1 antibody levels correlated with albumin (*p* = 0.013, r = 0.573), but no other correlation was observed between the measured antibodies, including anti-CMV immunoglobulin G (IgG) levels, and the available clinical parameters, including survival time, age, CRP, albumin, and VitD, in the patient cohort. No difference was detected in the antibody magnitudes against rhinovirus, HCoV-OC43, HCoV-HKU1, HCoV-229E, HCoV-NL63, EBV, influenza A (H1N1), or CMV, between end-of-life patients and elderly controls (Figure 1B), implying that there was no significant disparity in viral exposure between the cohorts. This suggests that established humoral immunity in end-of-life patients remains intact and comparable to elders of similar age range.

### Fewer central memory T cells in end-of-life patients compared to elderly healthy controls

To investigate if the bulk T cell populations, defined as CD3^+^ T cells among live CD14^−^CD19^−^ lymphocytes, were affected, as hypothesized, in end-of-life patients, compared to elderly controls, a high-parameter flow cytometry panel was designed for T cell phenotyping directly *ex vivo* (Figure S1C). Based on the expression of surface markers CCR7 and CD45RA, T cells may be characterized into naive T cells (T_NAIVE_; CCR7^+^CD45RA^+^), central memory T cells (T_CM_; CCR7^+^CD45RA^−^), effector memory T cells (T_EM_; CCR7^−^CD45RA^−^), and effector memory T cells re-expressing CD45RA (T_EMRA_; CCR7^−^CD45RA^+^). There was no difference in the number of CD3^+^ T cells, the CD4/CD8 ratio, or in the proportions of CD4^+^, CD8^+^, or naive T cells between patients and controls (Figures 1C–1F). The patients exhibited a significantly lower proportion of CD4^+^ and CD8^+^ T_CM_ compared to controls, but no difference was observed for T_EM_ and T_EMRA_ cells (Figure 1F). Besides the lower proportion of CD4^+^ and CD8^+^ T_CM_ in the end-of-life patients, the T cell compartment seems to maintain a similar composition at the end of life to that of healthy elders.

### Increased activation, but not exhaustion, in memory T cells at the end of life

Similarly to other bodily functions, aging is associated with progressive T cell senescence and exhaustion, which affects the ability of the T cells to respond to antigenic stimuli.^32^ Subsequently, the elderly are more prone to acquire infections and develop cancer. When comparing the expression of activation, exhaustion, and cytotoxic markers on the bulk CD4^+^ and CD8^+^ T cell subsets among live CD3^+^ lymphocytes, patients had significantly higher frequency of T cells expressing the classical activation markers CD38 and HLA-DR, compared to controls (Figures 1G, S1D, and S1E). CD4^+^ and CD8^+^ T_CM_ in the patient cohort exhibited higher expression of co-stimulatory marker CD226 (Figures 1G, S1D, and S1E). Meanwhile, the expression of TIGIT, the competitive, inhibitory receptor to CD226, was significantly decreased on the surface of CD4^+^ and CD8^+^ T_CM_, and on CD8^+^ T_EM_ and T_EMRA_ cells, in the patient cohort (Figures 1G and S1D). Upregulation of CD226 and downregulation of TIGIT are suggestive of activation rather than exhaustion. In agreement with this notion, expression of PD-1, a well-established inhibitory receptor, was only significantly upregulated on the patients’ T_EMRA_ cells, and downregulated on CD4^+^ T_NAIVE_ cells, compared to controls (Figures 1G and S1D). To further evaluate the concept of activated (CD38^+^, HLA-DR^+^, CD226^+^) versus the exhausted phenotypes (TIGIT^+^, PD-1^+^), where exhaustion is characterized by reduced proliferation, responsiveness, and functionality, we assessed the functionality of resting mT cells using the cytotoxic markers granzyme B (GzmB) and CD107a and the proliferative marker Ki-67. Patients’ CD4^+^ T_EMRA_ cells more commonly expressed Ki67 while exhibiting lower frequency of GzmB expression, compared to controls, but this was not observed in the mCD8^+^ T_EMRA_ cells or in other mCD4^+^ T cell subsets (Figures 1G and S1D). GzmB expression was, however, upregulated in the CD8^+^ T_EM_ from patients compared to controls (Figures 1G and S1D). Furthermore, only CD4^+^ T_CM_ cells exhibited a difference in the frequency of CD107a (Figures 1G and S1D). Our analysis also included 2B4 (alternatively known as CD244 or SLAMf4), a surface receptor associated with activation, inhibition, or cytotoxic activity, depending on the cell type and extracellular environment.^33^^,^^34^^,^^35^^,^^36^ In our dataset, there were strong correlations between the expression frequency of 2B4 and the production of the cytotoxic markers GzmB and CD107a, in both the mCD4^+^ and mCD8^+^ T cell compartments (Figures 1H and 1I). Additionally, 2B4^+^ T cell frequency correlated with the expression of the stimulatory markers HLA-DR and CD226, while negatively correlating or lacking correlation with the expression of the inhibitory markers TIGIT and PD-1 (Figures 1H and 1I). Based on this, 2B4, in our dataset, appeared to be predominantly associated with an activated phenotype with cytotoxic potential, especially in the mCD8^+^ T cell compartment. 2B4 was downregulated in the patients’ CD4^+^ T_EMRA_ cells, compared to controls (Figures 1G and S1D). Collectively, these results suggest that despite the elevated expression of activation markers on the end-of-life patients T cells, there is limited evidence for exhaustion or functional deficits.

### Increased frequency of cytotoxic mCD4^+^ T cells in the last month of life

**Figure 2:**
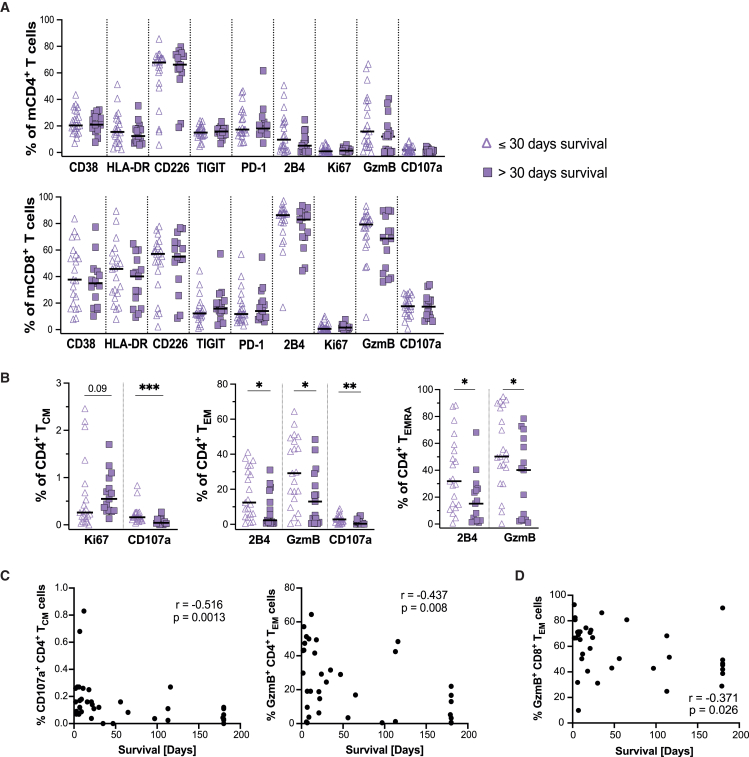
Increased frequency of cytotoxic mCD4+ T cells in the last month of life (A) Graph showing the frequency of cells expressing the specified marker among unstimulated mCD4+ T cells (left) and mCD8+ T cells (right) in end-of-life patients stratified for survival time. The end-of-life patient cohort was stratified into patients who survived less than 30 days post-sampling (*n* = 21) and those who survived more than 30 days (*n* = 15). Mann Whitney U test. (B) Graph showing the frequency of cells expressing the specified marker among the mCD4+ TCM, TEM, and TEMRA cells when stratified for a survival time, ≤30 days (*n* = 21) or >30 days (*n* = 15), post-sampling. Mann Whitney U test. (C) Spearman correlation between patient (*n* = 36) survival time in days and frequency of CD107a+CD4^+^ TCM (left) and GzmB+CD4^+^ TEM cells (right). (D) Spearman correlation between patient (*n* = 36) survival time in days and frequency of GzmB+ CD8^+^ TEM cells. Correlation graphs show spearman correlation coefficient (r) and *p* value (p). ∗*p* < 0.05; ∗∗*p* < 0.01; ∗∗∗*p* < 0.001; ∗∗∗∗*p* < 0.0001. The median (black line) is shown when applicable.

Since the deterioration of bodily functions is usually rapid during the last weeks of life,^1^^,^^37^ we divided our patient cohort based on survival time, where 30 days was used as the cutoff, to further assess signs of deterioration in the T cell compartment at the end of life. No difference was observed in the frequency of CD3^+^ T cells among live lymphocytes, bulk CD4^+^ and CD8^+^ T cells, or in the frequency of their naive or memory subsets (Figures S2A–S2C). Similarly, survival time had no observable impact on the expression of the measured phenotypic, proliferative, or cytotoxic markers when comparing bulk mCD4^+^ and mCD8^+^ T cells (Figure 2A). However, some of the markers were differentially expressed across the CD4^+^ T cell subsets (Figures 2B and S2D), relative to survival time. The cytotoxic markers CD107a and GzmB and cytotoxicity-related marker 2B4 were elevated on CD4^+^ T_CM_ (CD107a), T_EM_ (2B4, GzmB, CD107a), and T_EMRA_ (2B4, GzmB) in the last 30 days of life, compared to patients who survived longer than 30 days (Figure 2B). Furthermore, CD107a frequency among CD4^+^ T_CM_ cells and the frequency of GzmB^+^ CD4^+^ T_EM_ and CD8^+^ T_EM_ cells correlated with survival time in end-of-life patients (Figures 2C and 2D). A downregulation of PD-1 in CD4^+^ T_NAIVE_ cells, which also positively correlated with survival time, was also observed in the last 30 days of life (Figures S2D and S2E). In summary, the T cell compartment remains largely intact in the last 30 days of life but exhibits an increased frequency of mCD4^+^ T cells with cytotoxic potential compared to patients with longer survival time.

### Virus-specific mCD4^+^ T cell responses are maintained at the end of life

After an infection, mT cells recognizing antigens of the cleared pathogen will remain in circulation. These antigen-specific mT cells can be identified through their rapid upregulation of activation-induced markers upon reactivation. Subsequently, to gain detailed insights into specific functions and characteristics of individual T cell subsets, we undertook a detailed analysis of seasonal OC43- and CMV-specific mCD4^+^ and mCD8^+^ T cells after a 10-h stimulation with OC43- or CMV-derived peptide pools using flow cytometry.

**Figure 3:**
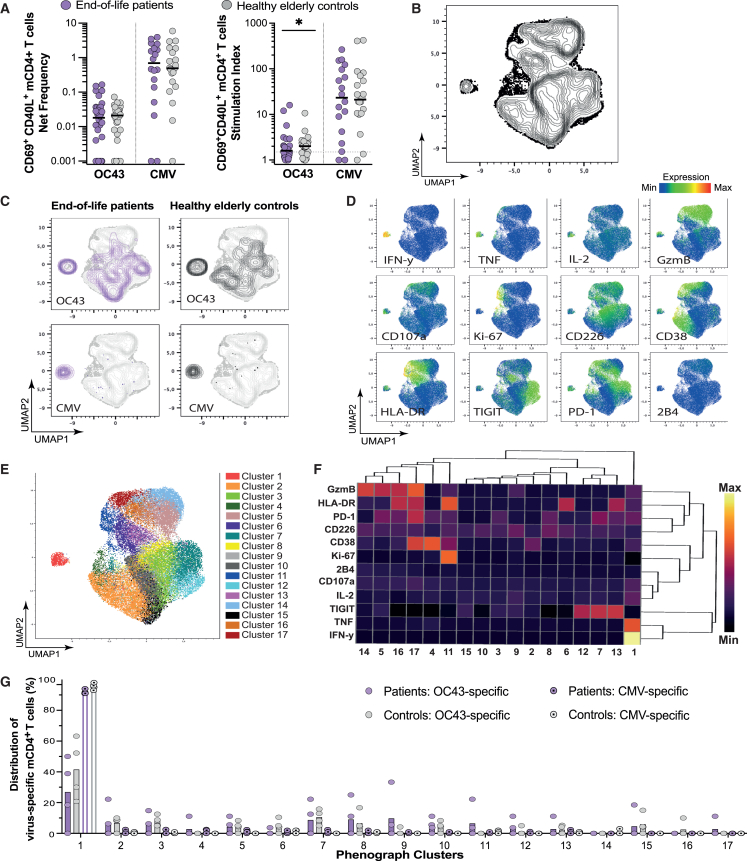
Virus-specific mCD4+ T cell responses are maintained at the end of life (A) Graphs showing the net frequency (left) and stimulation index (SI; right) of virus-specific mCD4+ T cells (CD69^+^CD40L+) in end-of-life patients (OC43, *n* = 34; CMV, *n* = 18) and elderly controls (OC43, *n* = 28; CMV, *n* = 20). Dashed line indicates the threshold of a positive response (SI > 1.5). Mann Whitney U-test. (B) UMAP contour of bulk and virus-specific mCD4+ T cells generated from four representative, positive donors for each condition and cohort (OC43 patients, *n* = 4; OC43 controls, *n* = 4; CMV patients, *n* = 4, CMV controls, *n* = 4). (C) UMAP contour (gray) overlayed with the distribution of virus-specific mCD4+ T cells in end-of-life patients (purple) and elderly controls (black). (D) UMAP plots overlayed with the median fluorescence intensity for each of the measured markers. (E) UMAP plot overlayed with clusters annotated with PhenoGraph based on the measured markers. (F) Heatmap showing the hierarchical clustering of median relative expression of the indicated markers in the clusters defined in (E). Data are represented as median relative expression. (G) Graph showing the median distribution (bar) of OC43-specific and CMV-specific mCD4+ T cells for end-of-life patients and elderly controls across the clusters shown in (E), with individuals shown as filled circles with (CMV) or without a dot (OC43). Mann Whitney U test.∗*p* < 0.05; ∗∗*p* < 0.01; ∗∗∗*p* < 0.001; ∗∗∗∗*p* < 0.0001. The median (black line) is shown when applicable.

No difference was observed in the net frequency of OC43- and CMV-specific mCD4^+^ T cells, defined as CD69^+^CD40L^+^, between patients and controls (Figure 3A). Based on the stimulation index (SI), however, significantly fewer OC43-specific mCD4^+^ T cells were observed in the patients, compared to elderly controls (Figure 3A). There was no correlation between antibody levels and the frequency of antigen-specific mCD4^+^ T cells (Figure S3A). There was a significant, albeit weak, negative correlation between age and the frequency of OC43-specific, but not CMV-specific, mCD4^+^ T cells (Figure S3B). Next, the expression of phenotypic and functional markers on the antigen-specific mCD4^+^ T cells were visualized using Uniform Manifold Approximation and Projection for Dimension Reduction (UMAP). OC43- or CMV-specific mCD4^+^ T cells, from representative patients diagnosed with cancer and controls with adequate cell numbers, were projected alongside bulk mCD4^+^ T cells (Figure 3B; Table S2; see STAR Methods for details). The distribution of OC43-specific or CMV-specific mCD4^+^ T cells in the generated UMAP was similar between end-of-life patients and elderly controls (Figure 3C). Notably, the UMAP revealed that OC43-specific T cell responses were scattered across the mCD4^+^ T cell population, whereas the CMV-specific mCD4^+^ T cells were more uniform in both cohorts (Figure 3C). The antigen-specific mCD4^+^ T cells clustered in areas associated with interferon gamma (IFN-γ), tumor necrosis factor (TNF), and interleukin-2 (IL-2) expression, which are the signature cytokines of the type 1 T helper (T_H_1) cells typically induced during viral infections (Figures 3C and 3D). Using PhenoGraph, 17 clusters were annotated based on the expression of measured markers (Figure 3E). The majority of the CMV-specific and a large proportion of most donor’s OC43-specific mCD4^+^ T cells were assigned to cluster 1, which demonstrated high expression of IFN-γ, TNF, and, to a lesser extent, IL-2, GzmB, and CD107a alongside the activation markers (Figures 3F and 3G). No significant difference was observed when comparing the distribution of cells in the clusters between patients and controls. Based on these results, antigen-specific mCD4^+^ T cells, induced by an acute (OC43) or chronic (CMV) infection, in end-of-life patients share similar characteristics to those of healthy elderly controls.

### Phenotypic changes to virus-specific CD4^+^ T cells have limited effect on functional capacity

**Figure 4:**
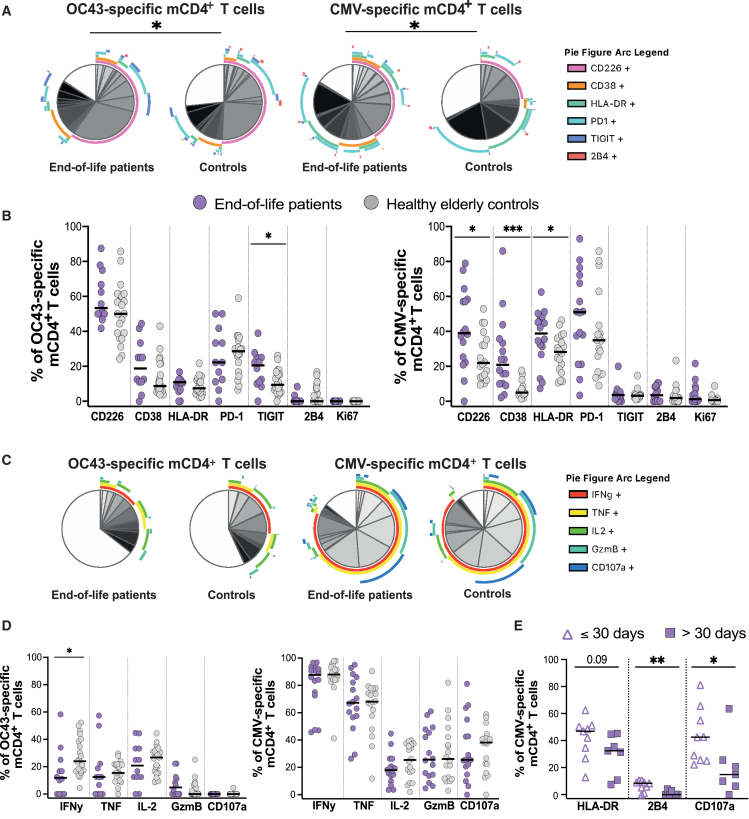
Phenotypic changes to virus-specific mCD4+ T cells have limited effect on functional capacity (A) Pie chart generated by SPICE showing the phenotypic profile of virus-specific mCD4+ T cells in end-of-life patients (OC43, *n* = 12; CMV, *n* = 16) and elderly controls (OC43, *n* = 21; CMV, *n* = 18). The white piece represents cells lacking expression of all included markers. Only donors with a positive antigen-specific response were included. Permutation test. Data are represented as mean frequency. (B) Graphs showing the frequency of virus-specific mCD4+ T cells from end-of-life patients (OC43, *n* = 12; CMV, *n* = 16) and elderly controls (OC43, *n* = 21; CMV, *n* = 18) expressing the measured phenotypic markers. Only positive donors were included. Mann Whitney U test. (C) Pie chart generated by SPICE showing the functional profile of virus-specific mCD4+ T cells in end-of-life patients (OC43, *n* = 12; CMV, *n* = 16) and elderly controls (OC43, *n* = 21; CMV, *n* = 18). The white piece represents cells lacking expression of all included markers. Only positive donors were included. Permutation test. Data are represented as mean frequency. (D) Graphs showing the frequency of virus-specific mCD4+ T cells in end-of-life patients (OC43, *n* = 12; CMV, *n* = 16) and elderly controls (OC43, *n* = 21; CMV, *n* = 18) expressing the measured functional markers. Only positive donors were included. Mann Whitney U test. (E) Graph showing the frequency of CMV-specific mCD4+ T cells expressing markers whose expression was, or close to, significantly different between end-of-life patients who survived ≤30 days (*n* = 9) or >30 days (*n* = 7) after sampling. Only positive donors were included. Mann Whitney U test.∗*p* < 0.05; ∗∗*p* < 0.01; ∗∗∗*p* < 0.001; ∗∗∗∗*p* < 0.0001. The median (black line) is shown when applicable.

To further explore the phenotype and function of virus-specific mCD4^+^ T cells, we used simplified presentation of incredibly complex evaluations (SPICE), a software to study the co-expression of markers from flow cytometry data (Figure S4A). There was a significant difference in the phenotypic profile for the OC43-specific mCD4^+^ T cell populations (Figure 4A), which appeared to be partly due to the increased frequency of cells expressing the inhibitory marker TIGIT among the patients, compared with controls (Figure 4B). Although no functional loss was observed when comparing the complete functional profile of OC43-specific mCD4^+^ T cells between the cohorts (Figure 4C), significantly fewer IFN-γ^+^ OC43-specific mCD4^+^ T cells were observed in the patients compared to controls (Figure 4D). Similarly to the OC43-specific mCD4^+^ T cells, the CMV-specific mCD4^+^ T cell populations displayed significant differences in their phenotypic profiles (Figure 4A), likely driven by the patients’ increased frequency of cells expressing the activation markers CD226, CD38, and HLA-DR (Figure 4B). Such phenotypic changes suggest increased activation in the CMV-specific mCD4^+^ T cell population at the end of life, yet there was no sign of alterations to their functional capacity, neither in their polyfunctional profile nor when investigating single marker frequency, between end-of-life patients and elderly controls (Figures 4C and 4D).

Since CMV infection and reactivation has previously been linked to frailty, cancer, and cardiovascular disease,^38^^,^^39^^,^^40^^,^^41^^,^^42^^,^^43^ we stratified the end-of-life patients based on survival time, with a cutoff at 30 days, to evaluate if CMV-specific mCD4^+^ T cells change at the end of life. The frequency of CMV-specific mCD4^+^ T cells was not significantly different between those who survived ≤30 or >30 days post-inclusion, although a trend for increased SI in the last 30 days was observed (Figure S4B). Neither was there a difference in their phenotypic or functional profiles (Figures S4C and S4D). At the single marker level, however, CMV-specific mCD4^+^ T cells from patients in their last 30 days of life had higher expression of the cytotoxic marker CD107a and cytotoxicity-related marker 2B4, compared to those who lived over 30 days (Figure 4E). Of note, both CD107a and 2B4 expression negatively correlated with survival time in this population (Figure S4E). OC43-specific mCD4^+^ T cells were also analyzed with regard to survival time but did not display any significant difference in the frequency nor in the phenotypic or functional profiles (Figures S4B–S4D), although a trend for a positive correlation between survival time and IFN-γ expression was noted (Figure S4E). These findings suggest that, irrespective of the frailty of the patient, virus-specific mCD4^+^ T cells largely maintain their functional capacity at the end of life.

### Virus-specific CD8^+^ T cells show a higher degree of activation in the end-of-life patient cohort

**Figure 5:**
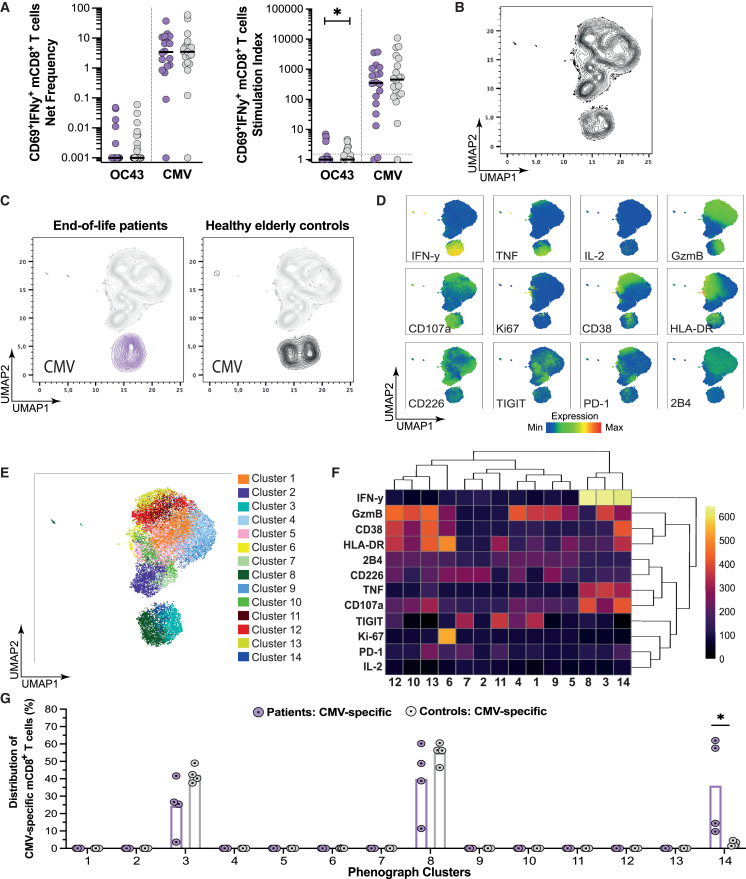
Virus-specific CD8^+^ T cells show a higher degree of activation in the end-of-life patient cohort (A) Graphs showing the net frequency (left) and stimulation index (right) of virus-specific mCD8+ T cells (CD69+IFN-γ+) in end-of-life patients (OC43, *n* = 33; CMV, *n* = 18) and elderly controls (OC43, *n* = 28; CMV, *n* = 20). Dashed line indicates the threshold of a positive response (SI ≥ 1.5). Mann Whitney U test. (B) UMAP contour of bulk mCD8+ T cells and CMV-specific mCD8+ T cells generated from four representative donors for each condition and cohort (patients, *n* = 4; controls, *n* = 4). (C) UMAP contour (gray) overlayed with the distribution of CMV-specific mCD8+ T cells in end-of-life patients (purple) and elderly controls (black). (D) UMAP plots overlayed with the median fluorescence intensity for each of the measured markers. (E) UMAP overlayed with clusters annotated with PhenoGraph based on the measured markers. (F) Heatmap showing the hierarchical clustering of median relative expression of the indicated markers in the clusters defined in (E). Data are represented as median relative expression. (G) Graph showing the median distribution (bar) of CMV-specific mCD8+ T cells within the clusters shown in (E), with individuals shown as filled circles with a dot. Mann-Whitney U test.∗*p* < 0.05; ∗∗*p* < 0.01; ∗∗∗*p* < 0.001; ∗∗∗∗*p* < 0.0001. The median (black line) is shown when applicable.

Virus-specific mCD8^+^ T cells, defined as CD69^+^IFN-y^+^, initially mirrored our findings in the CD4^+^ T cell compartment. A significant decrease in SI was observed for OC43-specific mCD8^+^ T cells in the patients, compared to the controls, but there was no difference in the net frequency of either OC43- or CMV-specific mCD8^+^ T cells between the cohorts (Figure 5A). Due to the low number of donors with OC43-specific mCD8^+^ T cells, only CMV-specific T cell responses were analyzed further. The frequency of CMV-specific mCD8^+^ T cells did not correlate with either anti-CMV IgG levels nor age (Figures S5A and S5B). The overall profile of CMV-specific mCD8^+^ T cells from representative donors, visualized using UMAP, displayed a high degree of uniformity between end-of-life patients and elderly controls, with nearly all CMV-specific mCD8^+^ T cells clustering with markers of activation, cytokine expression, and cytotoxicity (Figures 5B–5D). When looking at specific combinations of markers, 14 clusters were identified by PhenoGraph (Figure 5E). Notably, the patients had significantly more CMV-specific mCD8^+^ T cells in cluster 14, associated with activation markers CD38 and HLA-DR in addition to the functional markers (Figures 5F and 5G). These observations may suggest that although the CMV-specific mCD8^+^ T cell responses of the cohorts are similar, end-of-life patients displayed a more activated phenotype compared to healthy elderly living at home.

### CMV-specific mCD8^+^ T cell polyfunctionality and cytotoxicity increases at the end of life

**Figure 6:**
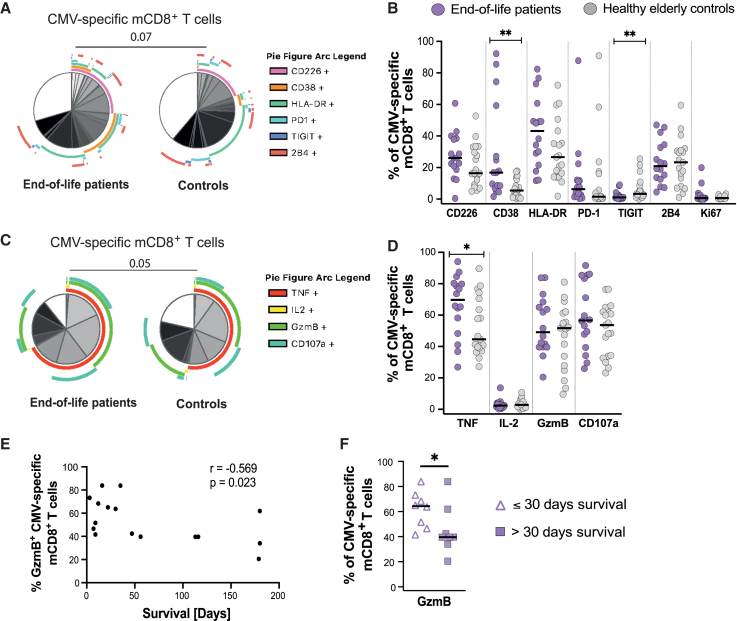
CMV-specific mCD8+ T cell polyfunctionality and cytotoxicity increases at the end of life (A) Pie chart generated by SPICE showing the phenotypic profile of CMV-specific mCD8+ T cells in end-of-life patients (*n* = 16) and elderly controls (*n* = 19). The white piece represents cells lacking expression of all included markers. Only donors with a positive CMV-specific response were included. Permutation test. Data are represented as mean frequency. (B) Graph showing the frequency of CMV-specific mCD8+ T cells expressing the measured phenotypical markers in end-of-life patients (*n* = 16) and elderly controls (*n* = 19). Only donors with a positive response were included. Mann Whitney U test. (C) Pie chart generated by SPICE showing the functional profile of CMV-specific mCD8+ T cells in end-of-life patients (*n* = 16) and elderly controls (*n* = 19). White piece represents cells lacking expression of all included markers. Only donors with a positive response were included. Permutation test. Data are represented as mean frequency. (D) Graph showing the frequency of CMV-specific mCD8+ T cells in end-of-life patients (*n* = 16) and elderly controls (*n* = 19) expressing the measured functional markers. Only donors with a positive response were included. Mann Whitney U test. (E) Spearman correlation between the frequency of GzmB+ CMV-specific mCD8+ T cells and survival time post-sampling. Only donors with a positive response were included. (F) Graph showing the frequency of GzmB+ CMV-specific mCD8+ T cells in end-of-life patients who survived ≤30 days (*n* = 8) or >30 days (*n* = 8) after sampling. Only donors with a positive response were included. Mann Whitney U test. Correlation graphs show spearman correlation coefficient (r) and *p* value (p). ∗*p* < 0.05; ∗∗*p* < 0.01; ∗∗∗*p* < 0.001; ∗∗∗∗*p* < 0.0001. The median (black line) is shown when applicable.

The composition of the CMV-specific mCD8^+^ T cell populations was further assessed using SPICE. A non-significant trend (*p* = 0.07) was observed between the phenotypic profile of CMV-specific mCD8^+^ T cells from patients and controls (Figure 6A), likely driven by the frequency of CD38 and TIGIT expression, which were significantly increased and decreased, respectively, in the patients’ CMV-specific mCD8^+^ T cells (Figure 6B). Similarly, the CMV-specific mCD8^+^ T cells exhibited a non-significant trend (*p* = 0.05) in their functional profiles when comparing the two cohorts (Figure 6C). When followed up at the single-marker level, it was revealed that CMV-specific mCD8^+^ T cells expressing TNF were significantly more common in patients than in controls (Figure 6D). Since the antigen-specific mCD8^+^ T cells were defined as CD69^+^IFN-γ^+^, the higher frequency of TNF co-expression in the patient cohort is analogous to increased polyfunctionality. Furthermore, GzmB expression negatively correlated with survival time (Figure 6E), where patients who lived 30 days or less after sampling had significantly more cytotoxic (GzmB^+^) CMV-specific mCD8^+^ T cells than those with longer survival time (Figure 6F). However, there was no difference in the phenotypical nor functional profiles based on survival time (Figures S6A and S6B). In summary, these findings stand in contradiction to our hypothesis that antigen-specific T-cell-mediated immunity would deteriorate at the end of life and may instead imply an increased activity at this stage.

## Discussion

Advanced age is a major risk factor associated with increased susceptibility to and more severe clinical outcomes in various viral infections.^12^^,^^13^^,^^14^ Previous research, including a study by Humbert et al., has shown that antigen-specific cellular immunity declines with age.^5^^,^^6^^,^^7^^,^^8^^,^^27^ Whether aging of the immune system is further exacerbated in the final days and weeks of life in older individuals is unknown. It has previously been shown that antibody titers against some viruses are lower in the elderly population.^44^ Here, we show that end-of-life patients exhibit comparable antibody levels to multiple viral antigens responsible for common acute upper respiratory infections (influenza A, rhinovirus, HCoV-OC43, -HKU1, -229E, and -NL63) and chronic, latent, herpesvirus infections (EBV and CMV), compared to elderly healthy controls. Our data corroborate that vaccine recommendations for adults 65 years and older is valid for end-of life patients.^45^ Furthermore, we observed no correlation between virus-specific T cell responses and corresponding antibody levels in our cohorts, which is in accordance with previous studies.^27^^,^^46^^,^^47^ This indicates that humoral and cellular immunity are independently maintained and that both arms of adaptive immunity must be studied to fully understand the maturation and decay of immune memory.

Frailty in older individuals has been depicted by biomarkers associated with inflammation, oxidative stress, skeletal/cardiac muscle function, and platelet function.^48^ Here, the end-of-life patients displayed higher levels of CRP and lower levels of albumin and VitD than the elderly controls. High CRP and low albumin levels are markers of inflammation and disease burden, especially in cancer, and are associated with poor prognosis.^49^ We previously observed that low VitD levels are common in patients receiving palliative care.^50^^,^^51^^,^^52^ Low levels of VitD have been shown to increase the risk for respiratory tract infections.^53^ Moreover, VitD receptor signaling was shown to suppress T-cell-mediated inflammation in the context of COVID-19, the response to VitD being impaired in severe cases.^54^ Our data support a heightened inflammatory response in the end-of-life patient cohort.

Given the limited research on adaptive cellular immunity (mT cells) at the end of life, we contextualized our findings relative to current literature on the relationship between T cells, frailty, and mortality in the elderly. A low (<1) CD4/CD8 ratio has been associated with an increased 2-year mortality,^55^^,^^56^ although this has been repeatedly contested.^57^^,^^58^^,^^59^ In the present study, no difference was detected in the CD4/CD8 ratio, number of CD3^+^ T cells, among live lymphocytes, CD4^+^ T cells, CD8^+^ T cells, naive, nor mT cell frequencies between patients and elderly controls. Similarly, no difference was detected in the CD4^+^ and CD8^+^ T_EM_ and T_EMRA_ cell frequencies between the study cohorts. However, end-of-life patients exhibited significantly fewer CD4^+^ and CD8^+^ T_CM_ cells compared to elderly controls. T_CM_ cells circulate between lymphoid tissues and exhibit high proliferative capacity but with limited effector functions, contrasting with the robust effector functions of the peripheral T_EM_ and T_EMRA_ cells.^60^ Due to their longevity and proliferative capacity, human and murine T_CM_ have been suggested to confer greater protective immunity in vaccine-induced, antiviral, and anti-tumoral responses than T_EM_.^61^^,^^62^^,^^63^^,^^64^^,^^65^ In cancer patients, high numbers of peripheral T_CM_ cells have been linked to a beneficial clinical outcome.^63^^,^^66^ Therefore, the patients’ reduced T_CM_ population could imply less efficient antigen clearance upon challenge. Possible explanations for this T_CM_ decline include suboptimal T_CM_ differentiation due to high antigen load and sustained inflammation at the end of life,^67^ as well as epigenetic alterations induced in aging T cell populations.^68^ Here, together with signs of inflammation, we detected higher levels of immune activation, based on the expression of CD38, HLA-DR, and CD226 on mT cells at the end of life. Alternatively, a higher percentage of T_CM_ may migrate to tissues at his life stage, leading to reduced peripheral T_CM_ counts.^69^

Antigen-specific mCD4^+^ T cell populations targeting two common viral infections, acute HCoV-OC43 and chronic latent CMV, and mCD8^+^ T cells targeting CMV were present at similar frequencies in end-of-life patients and elderly controls. As hypothesized, and as previously shown,^27^ the functional and phenotypic characteristics of the HCoV-OC43- and CMV-specific mCD4^+^ T cell populations were clearly distinguishable from each other, regardless of the cohort. Although the antigen-specific mCD4^+^ and mCD8^+^ T cells largely remained intact at the end of life, some differences were observed in comparison to elderly controls. Least sign of phenotypic difference was noted on the HCoV-OC43-specific mCD4^+^ T cells with the end-of-life patients, which only displayed a higher frequency of TIGIT. Across the CMV-specific mCD4^+^ and mCD8^+^ T cells, activation markers CD226, CD38, and HLA-DR were consistently upregulated in the patient cohort. CD226 is a costimulatory receptor competing for the same ligand as TIGIT,^70^^,^^71^ and CD38 is mostly known for its roles in cell metabolism and migration.^72^ CD38^+^HLA-DR^+^ mT cell frequencies have been shown to increase in chronic lymphocytic leukemia, gliomas, chronic infections, and immune disorders, where they are typically associated with a worse prognosis.^73^^,^^74^^,^^75^^,^^76^^,^^77^^,^^78^^,^^79^ In addition to the role of HLA-DR as an activation molecule, HLA-DR^+^ CD8^+^ T cells may serve a regulatory role alongside FOXP3^+^CD25^+^CD4^+^ T regulatory cells (Tregs).^80^ Since markers to identify Tregs or to evaluate regulatory functions of HLA-DR^+^ CD8^+^ T cells were not included in our panel, we can only speculate on their role at the end of life. Both Tregs and HLA-DR^+^ mCD8^+^ T cells accumulate with age, but, although the immunosuppressive capacity of Tregs appear stable, or even enhanced, the capacity of HLA-DR^+^CD8^+^ T cells decline with age.^81^^,^^82^ Treg accumulation in elders has been associated with cancer development and poor responses to influenza vaccination, presumably due to heightened immunosuppression.^83^^,^^84^ In summary, compared to that of elderly controls, CMV-specific mT cells from end-of-life patients exhibited signs of increased activation, for which one explanation may be that dysfunctional regulatory responses driven by age are further exacerbated by their condition.^76^ However, further research is needed to thoroughly explore this hypothesis.

PD-1 and TIGIT are markers of T cell exhaustion and immunosenescence,^85^^,^^86^^,^^87^ two parameters that reduce T cell responsiveness and function. TIGIT^+^CD8^+^ T cell frequency increases with age and has been suggested to reflect T cell senescence.^88^ Contrary to our initial hypothesis, CMV-specific mCD8^+^ T cells from end-of-life patients displayed lower TIGIT expression compared to controls. Accordingly, there was a higher frequency of TNF-expressing CMV-specific mCD8^+^ T cells in the patient cohort compared to controls. However, this was not observed for the CMV-specific mCD4^+^ T cells, where no difference was detected in either TIGIT^+^ or IFN-y^+^ cell frequency. On the other hand, we observed diminished frequency of IFN-y-expressing HCoV-OC43-specific mCD4^+^ T cells in end-of-life patients, compared to controls, which might be attributed to the higher frequency of TIGIT^+^ cells in this population. Overall, these results suggest that T cell exhaustion and immunosenescence do not uniformly affect all antigen-specific T cells investigated in our study. Specifically, T cells responding to acute (HCoV-OC43), rather than chronic (CMV), viruses appear to be disproportionately impacted by functional deficits at the end of life.

It is well documented that cytotoxic T cells accumulate with age and during acute and chronic viral infections.^89^ These cells play a crucial role in eliminating virally infected cells, where, for instance, GzmB production by CD8^+^ T cells was shown to predict the effectiveness of an influenza vaccine in older adults.^90^ There is also a growing interest in cytotoxic CD4^+^ T cells, due to their exceptionally high levels in supercentenarians (individuals over 110 years old),^91^ antitumor activity, and recently proposed role in the elimination of senescent cells in the skin.^92^ Given this context, it is intriguing to consider whether the surge of cytotoxic mCD4^+^ T cells (bulk, HCoV-OC43- and CMV-specific) we observed in the last 30 days of life is linked to increased cell senescence. Besides aging, cell senescence may be induced by cellular stress, such as DNA damage and CMV reactivation (reviewed in^93^). Considering the prevalence of CMV infection and reactivation in the immunocompromised and critically ill, and it being known to promote cytotoxic T cell accumulation,^39^^,^^40^^,^^43^^,^^94^ elevated CMV activity could be another cause for the high frequency of cytotoxic T cells in our end-of-life patients. However, no significant difference was observed in the anti-CMV IgG titers or CMV-specific T cell frequencies between these patients and elderly controls. This suggests a similar degree of CMV infection or reactivation, though it should be noted that CMV-specific immune responses do not necessarily correspond to CMV-derived antigen load nor to the level of viral activity.^95^ Although high anti-CMV titers are associated with increased frailty,^96^^,^^97^ the relationship between CMV-reactive T cells, mortality, and frailty is not completely understood. Studies have shown that a notable portion of T cells in CMV-seropositive elders recognize CMV-derived peptides—up to 40% of mT cells^98^^,^^99^—and that high frequencies of CMV-specific mCD4^+^ T cells correlate with an elevated risk of respiratory viral infections in nursing home patients.^81^ Additionally, research indicates that the CMV-specific mCD4^+^ T cell repertoire in elderly is dominated by low-avidity clones,^100^ whereas a declining CMV-specific mCD8^+^ T cell repertoire is linked to increased mortality.^101^^,^^102^ These findings raise questions about the ability of T cells to control CMV infection in old age. Future studies could explore the diversity of T cell clonotypes in end-of-life patients.

To our knowledge, this study represents the first in-depth exploration of antiviral, adaptive cellular immunity in end-of-life patients. Our results indicate durable cellular and humoral adaptive immunity against viral infections in these patients, when compared to older adults living at home. For physicians in palliative care, understanding that viral immunity largely remains intact at the end of life is valuable for potentially guiding trials aimed at improving clinical practice. For instance, targeted clinical trials designed to monitor vaccine-induced immunity against common viruses, such as SARS CoV-2, influenza, and varicella-zoster virus, could be considered for this vulnerable group.

### Limitations of the study

When stratifying the patient cohort by survival time, age distributions were similar between the two patient subgroups, making age an unlikely bias in this comparison. However, our patient cohort was significantly older than the control one, which could introduce a survival effect or a confounding impact of age. Although we observed limited differences in the viral immunity profiles between the patients and controls, our ability to exclude any potential bias induced by the age disparity was restricted because of the rather small sample size of our cohorts, preventing us from performing multivariable regression analysis that would adjust for age or comorbidities. The possibility that age-related differences could influence our findings should therefore be kept in mind when interpreting the present results, and we acknowledge that additional studies with age-matched patients and controls are necessary to validate our findings. Second, due to sample limitations and responsiveness, only T cell antigen specificity against two common viruses (HCoV-OC43 and CMV) were assessed in detail. Further research including additional viral antigens will be needed to determine whether our findings are applicable to a broader range of acute and chronic viruses. Considering that bacterial infections frequently lead to pneumonia and sepsis in patients receiving palliative care,^3^ future studies should investigate the function of antibacterial immunity at the end of life. Third, we focused on adaptive immunity; therefore, innate immunity in the context of end of life should also be explored. Due to the available material (peripheral blood mononuclear cells [PBMCs] and plasma), only peripheral antiviral immunity was assessed, which does not necessarily reflect the tissue-specific innate and adaptive immunity whose role in the prevention of infection and disease is becoming increasingly evident.^103^^,^^104^ Fourth, since we did not assess antibody functionality (neutralizing ability) nor vaccine-induced antibody responses, the clinical effectiveness of antibodies in end-of-life patients should be further studied. Lastly, the patient cohort is heterogeneous and includes both cancer and non-cancer patients with varying survival time, which complicates the interpretation of the findings. However, this reflects the actual clinical situation in palliative care where the patient cohorts are heterogeneous, with different diseases, different age, at different stages in their disease trajectory, and with unknown remaining survival time. Thus, the results described here can be applied to the actual clinical situation by realistically reflecting the target patient group. Although we believe our patient cohort is representative of patients in palliative care as a whole, we acknowledge that the diagnosis, treatment, and comorbidities of the patients included in our cohort will have influenced the results and that our findings may not be equally applicable to all patient subgroups in palliative care.

## Resources availability

### Lead contact

Further information and requests for resources should be directed to and will be fulfilled by the lead contact, Annika C. Karlsson (annika.karlsson@ki.se).

### Materials availability

This study did not generate new unique reagents.

### Data and code availability

• All data reported in this paper will be shared by the lead contact upon request.
• This paper does not report original code.
• Any additional information required to reanalyze the data reported in this paper is available from the lead contact upon request.

## Acknowledgments

We want to warmly thank all the patients and their families and the healthy volunteers for participating in this clinical study. We also want to thank research nurse Caritha Klasson for skillful work with collection of blood samples. This study was supported by grants from The Swedish Cancer Society (L.B.-B.: CAN2018/316), The Swedish Research Council (L.B.-B.: 2022-00651, A.C.K.: 2020-02033), Stockholm County Council (L.B.-B.: FoUI-974833), Centrum för Innovativ Medicin (CIMED) (L.B.-B., A.C.K.: FoUI-988737), and 10.13039/501100004047Karolinska Institutet (A.C.K.: 2022-01865, 2019-00931). Graphical abstract created with BioRender. Olofsson, A. (2024). https://BioRender.com/w49m444.

## Author contributions

Conceptualization, L.B.-B. and A.C.K.; methodology, M.H. and A.C.K.; investigation, A.O., M.H., and F.L.; formal analysis, A.O.; resources, R.S.R., H.M.F., L.B.-B., and A.C.K.; writing—original draft, A.O., P.B., L.B.-B., and A.C.K.; writing—review & editing, A.O., M.H., P.B., L.B.-B., and A.C.K.; visualization, A.O.; supervision, P.B., L.B.-B., and A.C.K.; funding acquisition, L.B.-B. and A.C.K. A.O. and A.C.K. had unrestricted access to all data.

All authors have agreed to submit the manuscript, read, and approved the final draft and take full responsibility of its content, including the accuracy of the data and the fidelity of the trial to the registered protocol and its statistical analysis.

## Declaration of interests

The authors declare no competing interests.

## STAR★Methods

### Key resources table

**Table:** 

REAGENT or RESOURCE	SOURCE	IDENTIFIER
**Antibodies**		

UCHT1 (BUV805) [anti-CD3]	BD Biosciences	Cat#612895; RRID: AB_2870183
741182 (BUV737) [anti-TIGIT]	BD Biosciences	Custom conjugate
FN50 (BUV563) [anti-CD69]	BD Biosciences	Cat#748764; RRID: AB_2873167
SK3 (BUV496) [anti-CD4]	BD Biosciences	Cat#612936; RRID: AB_2870220
RPA-T8 (BUV395) [anti-CD8]	BD Biosciences	Cat#563795; RRID: AB_2722501
DX11 (BV750) [anti-CD226]	BD Biosciences	Cat#747026; RRID: AB_2871797
G46.6 (BV605) [anti-HLA-DR]	BD Biosciences	Cat#562844; RRID: AB_2744478
M5E2 (V500) [anti-CD14]	BD Biosciences	Cat#561391; RRID: AB_10611856
HIB19 (V500) [anti-CD19]	BD Biosciences	Cat#561121; RRID: AB_10562391
GB11 (BB790-P) [anti-Granzyme B]	BD Biosciences	Custom conjugate
HIT2 (BB700) [anti-CD38]	BD Biosciences	Cat#566445; RRID: AB_2744375
B56 (Alexa Fluor 647) [anti-Ki67]	BD Biosciences	Cat#558615; RRID: AB_647130
C1.7 (FITC) [anti-2B4] (anti-CD244)	Biolegend	Cat#329506; RRID: AB_1279186
MQ1-17H12 (PE-Dazzle594) [anti-IL-2]	Biolegend	Cat#500344; RRID: AB_2564091
B27 (PE) [anti-IFN-g]	Biolegend	Cat#506507; RRID: AB_315440
G043H7 (APC-Cy7) [anti-CCR7] (anti-CD197)	Biolegend	Cat#353212; RRID: AB_10916390
H4A3 (BV785) [anti-CD107a] (anti-LAMP-1)	Biolegend	Cat#328644; RRID: AB_2565968
EH12.2H7 (BV711) [anti-PD-1] (anti-CD279)	Biolegend	Cat#329928; RRID: AB_2562911
HI100 (BV570) [anti-CD45RA]	Biolegend	Cat#304132; RRID: AB_2563813
24-31 (BV421) [anti-CD40L] (anti-CD154)	Biolegend	Cat#310824; RRID: AB_2562721
Mab11 (BV650) [anti-TNF]	Thermofisher Scientific	Cat#25-7349-82; RRID: AB_469686
LIVE/DEAD Fixable Aqua Dead Cell Stain Kit	Thermofisher Scientific	Cat#L34957
R-PE-conjugated Anti-human IgG Fc	Jackson Immunoresearch	RRID: AB_2922837
R-PE-conjugated monoclonal Anti-human IgG1	Southern Biotechnology	Cat#9054–09
Digoxigenin-conjugated human ACE2	Tran, T.T. et al.^105^	N/A
Monoclonal Anti-digoxigenin	Jackson Immunoresearch	RRID: AB_2339005

**Biological samples**		

Elderly adults living at home, >60 years of age	Stockholms Sjukhem, Palliative Medicine	N/A
Patients in palliative care, >60 years of age	Stockholms Sjukhem, Palliative Medicine	N/A

**Chemicals, peptides, and recombinant proteins**		

Foxp3/TF Staining buffer Set	Thermofisher Scientific	Cat#00-5523-00
BD GolgiPlug (with Brefeldin A)	BD Biosciences	Cat#555029
BD GolgiStop (with Monensin)	BD Biosciences	Cat#554724
Brilliant Stain Buffer Plus	BD Biosciences	Cat#566385
NeutrAvidin Protein	Thermofisher Scientific	Cat#31000
sulfo-LC-NHS-biotin	ProteoChem	Cat#b2103
Sodium Azide	VWR	Cat#786–299
D-biotin	Sigma-Aldrich	Cat#B4501
SARS-CoV-2 full length spike protein antigen	Hsieh, C.L. et al.^106^	N/A
SARS-CoV-2 RBD domain antigen	Amanat, F. et al.^107^	N/A
SARS-CoV-2 Nucleocapsid antigen	Prospec Bio	
HCoV-OC43 full length spike protein antigen	Sino Biologicals	Cat#40607-V08B
HCoV-HKU1 S1 antigen	Becker, M. et al.^108^	N/A
HCoV-229E S1 antigen	Becker, M. et al.^108^	N/A
HCoV-NL63 S1 antigen	Becker, M. et al.^108^	N/A
Rhinovirus antigen	Shrock, E. et al.^109^	N/A
EBV antigen	Shrock, E. et al.^109^	N/A
Influenza H1N1 antigen	Grødeland, Institute of Immunology, Oslo University Hospital.	2009 Swine Flu
CMV antigen	Roche Diagnostics	
Staphylococcal enterotoxin B	Sigma-Aldrich	S4881
Synthetic overlapping HCoV-OC43 Spike 20-mer peptides	Humbert, M. et al.^27^	N/A
Optimal CMV peptide pool (MHC-I and MHC-II)	Sette, La Jolla Institute for Immunology	N/A

**Software and algorithms**		

GraphPad Prism 9.5.1	Graphpad Software Inc.	https://www.graphpad.com/
SPICE 6.1	Roederer, M. et al.^110^	https://niaid.github.io/spice/
FlowJo 10.9.0	FlowJo, LLC	https://www.flowjo.com/
UMAP 3.3.3 and 4.1.1	FlowJo, LLC	https://www.flowjo.com/
DownSample 3.3.1	FlowJo, LLC	https://www.flowjo.com/
ClusterExplorer 1.7.6	FlowJo, LLC	https://www.flowjo.com/
Phenograph 4.0.3	FlowJo, LLC	https://www.flowjo.com/
RStudio 4.2.1	RStudio	https://www.rstudio.com/

### Experimental model and study participant details

#### Ethical considerations

Ethical permit, approving all the experiments and confirming that all experiments conform to the relevant regulatory standards, was granted by the Swedish Regional Ethical Review Authority in Stockholm (Dnr 2017/203-31/4). The study was conducted in accordance with the Helsinki Declaration and written informed consent was obtained from all participants.

#### Cohort characteristics

Venous blood samples were collected from patients (*n* = 38) at the specialized palliative in-patient ward at ASIH Stockholm South, Stockholm, Sweden in 2017 and 2020, with the last samples collected in October 2020. To investigate the impact of survival time, the patient cohort was later stratified based on survival time post sampling (≤30 days, *n* = 22; >30 days, *n* = 16). See Tables 1 and S1 for clinical parameters and sampling details. ASIH Stockholm South is a home care unit for supportive and palliative care that includes an in-patient ward with 16 beds for specialized palliative care in end-of-life patients. The average care time is 10–14 days and the majority (90%) of the patients suffer from advanced cancer. Details about the unit has been described earlier.^111^ None of the included patients died from an infection nor had an ongoing acute infection at the time of inclusion. Cause of death for all patients with cancer was their cancer disease according to the death certificates. Likewise, all patients with COPD, heart failure and ALS died due to these diseases. For two patients with COPD, pneumonia was stated as “contributing cause to death”. Elderly volunteers living at home were recruited to the control group (*n* = 28) through an advertisement at Karolinska Institutet, Stockholm, Sweden, and samples were collected by a study nurse from the palliative care unit. A separate dataset generated from the elderly control samples has been reported previously.^27^

### Method details

#### Plasma biomarkers

CRP (mg/L) and albumin (g/L) levels were analyzed by Karolinska University Laboratory at Karolinska University Hospital, Sweden, according to routine clinical practice.^112^ Vitamin D was measured as 25-hydroxyvitamin D (25-OHD) in serum using chemiluminesence immunoassay (CLIA) on the LIAISON instrument (DiaSorin Inc, Stillwater, MN, USA). The detectable range was 7.5 ± 175 nmol/L.

#### Isolation and cryopreservation of peripheral blood mononuclear cells and plasma

Peripheral blood mononuclear cells (PBMCs) were isolated from whole blood, collected with ethylenediaminetetraacetic acid (EDTA) or lithium-heparin as anticoagulant, using density gradient centrifugation with ficoll (Ficoll-Paque PLUS, Cytiva) according to the manufacturer’s instructions. The isolated PBMCs were then washed and cryopreserved in freezing medium constituted by fetal bovine serum (FBS; Sigma-Aldrich) with 10% dimethyl sulfoxide (DMSO; Sigma-Aldrich) at a concentration of 10–20 million cells/ml, and stored in liquid nitrogen until use. EDTA plasma was collected at the same time from a subset of samples and stored at −80°C.

#### Serology

Antibodies (IgG and IgG subtype 1) to viral antigens were measured with a multiplexed bead-based assay as described in detail elsewhere.^27^^,^^113^ In brief, plasma diluted 1:100 in assay buffer (PBS 1% Tween 20, 10 μg/ml D-biotin, 10 μg/mL Neutravidin, 0.1% Sodium Azide) was incubated for 30 min at 22°C with fluorescently-barcoded polymer beads coupled with neutravidin (ThermoFisher) and biotinylated viral antigens (sulfo-LC-NHS-biotin, ProteoChem, USA). The following antigens were included in the present assay: SARS-CoV-2, i.e., full-length (FL) spike protein, receptor-binding domain (RBD), nucleocapsid, human coronaviruses (HCoVs; S1 domains from HKU1, 229E and NL63, FL spike proteins from OC43 and NL63), EBV, Rhinovirus, and Influenza virus H1N1 (key resources table). The beads were washed thrice with PBS 1% Tween 20 (PBT) and labeled with R-PE-conjugated anti-human IgG Fc (Jackson Immunoresearch) before analysis by flow cytometry (Attune Nxt, ThermoFisher). The median R-PE fluorescent intensity (MFI) for each bead subset was divided by the background, determined by the MFI on beads coupled with only neutravidin. The threshold for a positive antibody response to SARS-CoV-2was set to a fold-change of 5 relative to background, which was based on measured serum antibody levels from hospitalized patients, ages 0–95 years. The results do not represent antibody titers per mL material, and levels are not comparable across viruses. Participants were not excluded from further analysis based on SARS-CoV-2 serostatus.

Detection of IgG-antibodies against cytomegalovirus (CMV) were conducted by the laboratory for clinical virology at the Karolinska University Laboratory, Stockholm, Sweden. The Cobas 8000 instrument (Roche Diagnostics) was used to quantify CMV IgG antibodies in human plasma using a CMV IgG kit (Roche Diagnostics) and electrochemiluminescence-analysis (ECLIA). The range was 0.25–500 U/ml and a value ≥0.5 U/ml was considered positive, which is in agreement with the manufacturer’s recommendations.

#### Peptides

20-mer peptides, overlapping by 10 amino acids, spanning the spike (S) protein of HCoV-OC43 were designed based on a consensus sequence generated from full genome metadata for OC43 publicaly available on January 27, 2020. One sequence per country, per year (human isolates only, no passaged isolates or isolates from vaccine development), were randomly selected to create a set of representative sequences for OC43. The sequences were downloaded from the NCBI GenBank and aligned using MAFFT, with the ‘-auto’setting, from which the consencus sequence was generated using the BioPython ‘dumb_concensus()’ method with default settings. The constituent peptides (*n* = 136) were synthesized by Sigma-Aldrich and are listed elsewhere.^27^ The lyophilized peptides were reconstituted in DMSO (Sigma-Aldrich) before being pooled to equal concentration in PBS to create the HCoV-OC43 spike peptide pool. Experimentally defined MHC-I-restricted 9–14 mer and MHC-II-restricted 15-mer peptides, overlapping by 10 amino acids, derived from CMV were obtained as a courtesy from Alessandro Sette’s group at La Jolla Institute for Immunology, CA, USA.^114^^,^^115^

#### Flow cytometry

Cryopreserved PBMCs were thawed at 37°C, washed and plated as one million cells per well in 100 μL RPMI 1640 (Sigma-Aldrich) supplemented with 10% Fetal Bovine Serum (FBS; Sigma-Aldrich) and 1% Penicillin/Streptomycin; Thermofisher). Cells were rested for at least 3h at 37°C with 5% CO_2_ and 10 U DNase (Roche) before stimulation with the HCoV-OC43 S-derived peptide pool (1 μg/mL) or with MCH-I and MCH-II-restricted peptides from CMV (0.5 μg/mL of each). Final DMSO concentration with cells was 0.1%. Cells were stimulated for 10 h at 37°C with 5% CO2. Brefeldin A (BD Bioscience), monensin (BD Bioscience) and anti-CD107a antibody were added 1 h after the stimulant. Positive and negative controls consisted of 0.5 μg/mL Staphylococcus enterotoxin B (SEB; Sigma Aldrich) and PBS with 0.1% DMSO (Sigma-Aldrich), respectively. Prior to antibody staining, cells were washed with FACS buffer (PBS containing 1% FBS and 2mM EDTA). Chemokine staining with anti-CCR7 was performed at 37°C for 10 min, followed by extracellular staining with Brilliant Violet Buffer (BD) and surface markers, including anti-CD4, anti-CD8, anti-CD45RA, anti-2B4, anti-PD-1, anti-HLA-DR, anti-CD226, anti-CD38, anti-CD14, anti-CD19, anti-TIGIT and viability dye, for 30 min in room temperature. The cells were fixed for 30 min at room temperature using the Foxp3 transcription factor fixation/permeabilization set (Invitrogen) prior to intracellular staining for 30 min at room temperature with Brilliant Violet Buffer and antibodies against anti-IFN-γ, anti-TNF, IL-2, anti-Granzyme B, anti-CD40L, anti-CD69, anti-41BB, anti-Ki67 and anti-CD3. Cells were washed with permeabilization buffer and resuspended in 1% paraformaldehyde before acquisition with the FACSymphony A5 (BD Biosciences) flow cytometer. This approach, short-term stimulation with peptide pools, enabled us to identify low frequency HCoV-OC43 and CMV-specific mCD4^+^ and mCD8^+^ T cells without prior knowledge of immunodominant peptides or the donors’ human leukocyte antigen (HLA)-type.

See Figure S1C for gating strategy. In brief, irregular flow events were excluded using a time gate. The lymphocytes and single cells were gated based on the SSC and FCS parameters. CD3^+^ T cells were then gated against the dump channel, consiting of LiveDead stain, anti-CD14 and anti-CD19. CD4^+^ and CD8^+^ T cells were gated out among live, CD14^−^CD19^−^ CD3^+^ lymphocytes. Naive and memory subsets were gated from the CD4^+^ and CD8^+^ T cell populations based on the expression of CCR7 and CD45RA. Naive T cells were excluded from analysis and antigen-specific T cells were identified in the memory (m) populations of CD4^+^ and CD8^+^ T cells as CD69^+^CD40L^+^ and CD69^+^IFNy^+^, respectively, after stimulation. Phenotypic and functional markers were gated on bulk memory populations before application to memory subsets and antigen-specific CD4^+^ or CD8^+^ T cells. Two end-of-life patients were excluded from all flow cytometry analyses due to low cell numbers. Results are shown as both net frequency, defined as the frequency of antigen-specific T cells after subtraction of the background observed in negative control, and as stimulation index (SI), defined as fold change of antigen-specific T cells relative to background in negative control. An SI ≥ 1.5 was considered a positive response and only donors with a positive response were used for downstream analysis of the antigen-specific T cell populations. Simplified presentation of incredibly complex evaluation (SPICE) was used to explore the combination of phenotypical or functional markers on the antigen-specific T cell populations.

#### Uniform manifold approximation and projection for dimension reduction (UMAP)

A subset of the bulk mCD4^+^ or mCD8^+^ T cell pool (*n* = 3000 downsampled using DownSample plugin v3.3.1) and antigen-specific mCD4^+^ or mCD8^+^ T cells (*n* ≤ 100, downsampled if needed) were concatenated so that each condition (HCoV-OC43 and CMV) was represented by four end-of-life patients and four elderly controls. Donor selection was restricted to those with a positive response to the analyzed antigen. Among those with a positive antigen response, donors were further selected based on their SI, where the median SI for the selected donors were made to match the median SI of their originated cohort (end-of-life patients or elderly controls) as closely as possible for each antigen. The end-of-life patients selected for the UMAP analysis had different cancer diagnoses. See Table S2 providing clinical data for end-of-life patients and elderly controls used to generate the UMAPs. The UMAPs were generated using the UMAP Flowjo plugin, v3.3.3 and v4.1.1, with default settings (Euclidean distance; nearest neighbors: 15; minimum distance: 0.5). Phenotypically related cells were clustered and explored using Phenograph v.4.0.3, k = 60, and ClusterExplorer v.1.7.6.

### Quantification and statistical analysis

Statistical analysis was performed with Graphpad Prism version 9.5.1 (Graphpad Software Inc.), R studio version 4.2.1, SPICE 6.1 and Flowjo version 10.8.1 (LLC). Normal distribution was assessed using the Shapiro-Wilk normality test. In case of normal distribution, parametric tests such as the unpaired T-test (2 groups) was used for significance testing. Unpaired non-parametric data was evaluated with the Mann Whitney U test (2 groups). Statistical analysis in SPICE was performed using the Permutation test with 10 000 iterations. Correlation analyses were done with Spearman correlation test, with the correlation coefficient and *p*-value, denoted as r and p respectively, shown in the graphs. The applied statistical test is specified in the corresponding figure legend. Statistical significance was defined as *p* < 0.05 (∗), *p* < 0.01 (∗∗), *p* < 0.001 (∗∗∗) and *p* < 0.0001 (∗∗∗∗). Unless otherwise specified, figures show the median when applicable.
