# Supplementary material for: Adaptive immune responses against common viruses are sustained and functional in end-of-life patients
Source: iScience. 2025 Feb 21;28(3):112082. doi: 10.1016/j.isci.2025.112082 (PMC11930376; doi:10.1016/j.isci.2025.112082)
Supplement: Document S1. Figures S1–S6 and Tables S1 and S2 [file mmc1.pdf]

iScience, Volume *28*

Supplemental information

Adaptive immune responses against common viruses are sustained and functional in end-of-life patients

Anna Olofsson, Marion Humbert, Rokeya S. Rekha, Maria Helde Frankling, Fridtjof Lund-Johansen, Peter Bergman, Linda Björkhem-Bergman, and Annika C. Karlsson

Table S1: Comorbidities for the end-of-life patients, related to Table 1.

	Diabetes Mellitus	Heart failure	Kidney failure	COPD
**Non-cancer condition as main diagnosis**				
COPD (n=4)	0	3	2	NA
Heart failure (n=2)	2	NA	1	0
ALS (n=1)	0	0	0	0
**Cancer as main diagnosis**				
Lung cancer (n=6)	2	1	0	2
GI-cancer (n=7)	1	1	0	0
Prostate, breast, gynecological cancer (n=8)	1	1	1	0
Hematological cancer (n=2)	0	0	0	0
Other (n=8)	1	0	0	0

COPD=Chronic Obstructive Pulmonary Disease

Table S2. Clinical data for end-of-life patients and elderly controls used to generate the UMAPs, related to Figure 3 and 5.

	**HCoV-OC43-specific $mCD4^+$ T cells**	**CMV-specific $mCD4^+$ T cells**	**CMV-specific $mCD8^+$ T cells**
**End-of-life patients**			
Donors for UMAP, n	4	4	4
Age, median in years	75.0	80.5	75.5
Survival time in days from inclusion, median (range)	14 (2-56)	15.5 (3-30)	10.5 (9-22)
**Healthy elderly controls**			
Donors for UMAP, n	4	4	4
Age, median in years	78.5	74.0	69.5

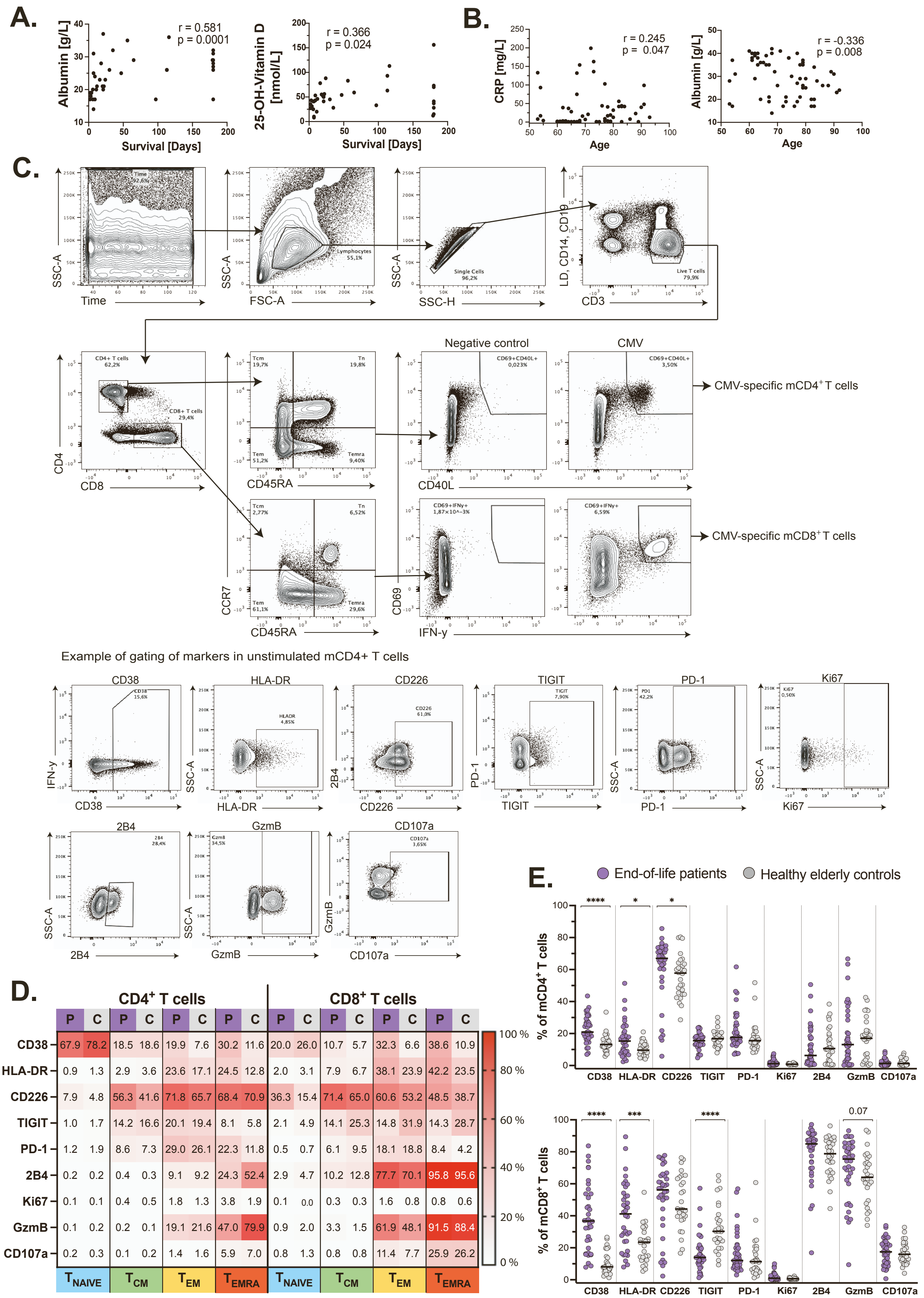
A.
Albumin [g/L]
Survival [Days]
r = 0.581
p = 0.0001
25-OH-Vitamin D [nmol/L]
r = 0.366
p = 0.024
B.
CRP [mg/L]
Age
r = 0.245
p = 0.047
r = -0.336
p = 0.008
C.
SSC-A
Time
FSC-A
Lymphocytes 55,1%
SSC-H
Single Cells 96,2%
LD, CD14, CD19
CD3
Live T cells 79,9%
CD4+ T cells 62,2%
CD8+ T cells 29,4%
CD4
CD8
CD45RA
CCR7
Tcm 19,7%
Tn 19,8%
Tem 51,2%
Temra 9,40%
Tcm 2,77%
Tn 6,52%
Tem 61,1%
Temra 29,6%
Negative control
CMV
CD69+CD40L+ 0,023%
CD69+CD40L+ 3,50%
CD40L
CMV-specific mCD4+ T cells
CD69+IFNy+ 1,87x10^-3%
CD69+IFNy+ 6,59%
CD69
IFN-y
CMV-specific mCD8+ T cells
Example of gating of markers in unstimulated mCD4+ T cells
CD38
HLA-DR
CD226
TIGIT
PD-1
Ki67
2B4
GzmB
CD107a
D.
CD4+ T cells
CD8+ T cells
P C P C P C P C P C P C P C P C
CD38 67.9 78.2 18.5 18.6 19.9 7.6 30.2 11.6 20.0 26.0 10.7 5.7 32.3 6.6 38.6 10.9
HLA-DR 0.9 1.3 2.9 3.6 23.6 17.1 24.5 12.8 2.0 3.1 7.9 6.7 38.1 23.9 42.2 23.5
CD226 7.9 4.8 56.3 41.6 71.8 65.7 68.4 70.9 36.3 15.4 71.4 65.0 60.6 53.2 48.5 38.7
TIGIT 1.0 1.7 14.2 16.6 20.1 19.4 8.1 5.8 2.1 4.9 14.1 25.3 14.8 31.9 14.3 28.7
PD-1 1.2 1.9 8.6 7.3 29.0 26.1 22.3 11.8 0.5 0.7 6.1 9.5 18.1 18.8 8.4 4.2
2B4 0.2 0.2 0.4 0.3 9.1 9.2 24.3 52.4 2.9 4.7 10.2 12.8 77.7 70.1 95.8 95.6
Ki67 0.1 0.1 0.4 0.5 1.8 1.3 3.8 1.9 0.1 0.0 0.3 0.3 1.6 0.8 0.8 0.6
GzmB 0.1 0.2 0.2 0.1 19.1 21.6 47.0 79.9 0.9 2.0 3.3 1.5 61.9 48.1 91.5 88.4
CD107a 0.2 0.3 0.1 0.2 1.4 1.6 5.9 7.0 0.8 1.3 0.8 0.8 11.4 7.7 25.9 26.2
TNAIVE TCM TEM TEMRA TNAIVE TCM TEM TEMRA
100 % 80 % 60 % 40 % 20 % 0 %
E.
End-of-life patients
Healthy elderly controls
% of mCD4+ T cells
% of mCD8+ T cells
****
*
*
***
****
0.07

Fig. S1. Expression of phenotypic and functional markers on bulk mT cell populations, related to Figure 1.

A. Spearman correlation between survival time and the plasma concentration of albumin and VitD in the end-of-life patient cohort (n = 38).

B. Spearman correlation between age and the plasma concentration of CRP and albumin across both cohorts (n = 62).

C. Gating strategy for the analysis of $CD3^+$ T cells among live $CD14^-CD19^-$ lymphocytes, and antigen-specific $mCD4^+(CD69^+CD40L^+)$ and $CD8^+$ $(CD69^+IFN\text{-}\gamma^+)$ T cells. Antigen-specific $mCD4^+$and $mCD8^+$ T cells, represented here by CMV-specific mT cells, were gated on the memory populations after exclusion of naïve T cells (T_N). Bottom plots show example gating of markers on resting $mCD4^+$ T cells.

D. Heatmap showing the median frequency of the indicated markers among the unstimulated $CD4^+$ and $CD8^+$ T cell subsets. Shown for end-of-life patients (P; n = 36) and elderly controls (C; n = 28), where the numbers indicate the median frequency. Results of significance testing are shown in Fig. 1G. T_{Naive}, naïve T cells; T_{CM}, central memory T cells; T_{EM}, effector memory T cells; T_{EMRA}, effector memory T cells re-expressing CD45RA. Mann Whitney U test. Data are represented as median frequency.

E. Graphs showing the frequency of unstimulated, bulk $mCD4^+$ (top) and $mCD8^+$ (bottom) T cells expressing the measured markers in end-of-life patients (n = 36) and elderly controls (n = 28). Mann Whitney U test.

Correlation graphs show spearman correlation coefficient (r) and p-value (p). *, $p < 0.05$; **, $p < 0.01$; ***, $p < 0.001$; ****, $p < 0.0001$. The median (black line) is shown when applicable.

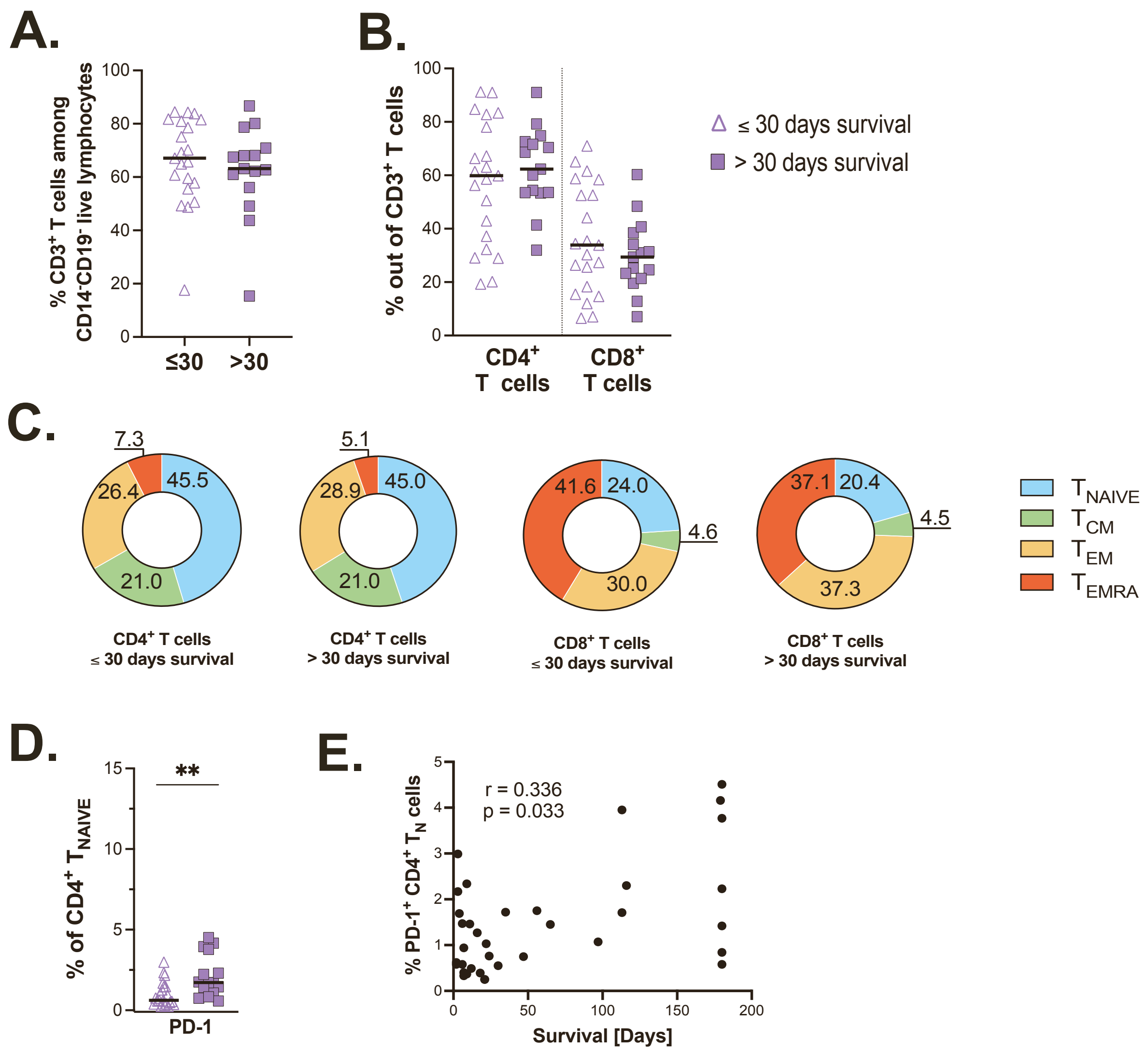

Fig. S2. The T cell compartment remains largely intact in the last month of life, related to Figure 2.

A. Graph showing the frequency of $CD3^+$ T cells among live $CD14^-CD19^-$ lymphocytes in end-of-life patients stratified for ≤ 30 days (n = 21) or > 30 days (n = 15) of survival after sampling. Mann Whitney U test.

B. Graph showing the frequency of $CD4^+$ T cells and $CD8^+$ T cells among $CD3^+$ T cells in end-of-life patients stratified for a survival time ≤ 30 days (n = 21) or > 30 days (n = 15) after sampling. Mann Whitney U test.

C. Donut graphs showing the mean frequency of naïve and memory T cell subsets among $CD4^+$ or $CD8^+$ T cells in end-of-life patients stratified for survival time (≤ 30 days, n = 21; > 30 days, n = 15). T_{Naive}, naïve T cells; T_{CM}, central memory T cells; T_{EM}, effector memory T cells; T_{EMRA}, effector memory T cells re-expressing CD45RA. Permutation test. Data are represented as mean frequency.

D. Graph showing the frequency of $PD1^+$ $CD4^+$ T_{NAIVE} cells in end-of-life patients that survived ≤ 30 days (n = 21) or > 30 days (n = 15) after sampling. Mann Whitney U test.

E. Spearman correlation between the frequency of $PD\text{-}1^+$ $CD4^+$ $T_{NAÏVE}$ cells and days of survival after sampling.

Correlation graphs show spearman correlation coefficient (r) and p-value (p). *, $p < 0.05$; **, $p < 0.01$; ***, $p < 0.001$; ****, $p < 0.0001$. The median (black line) is shown when applicable.

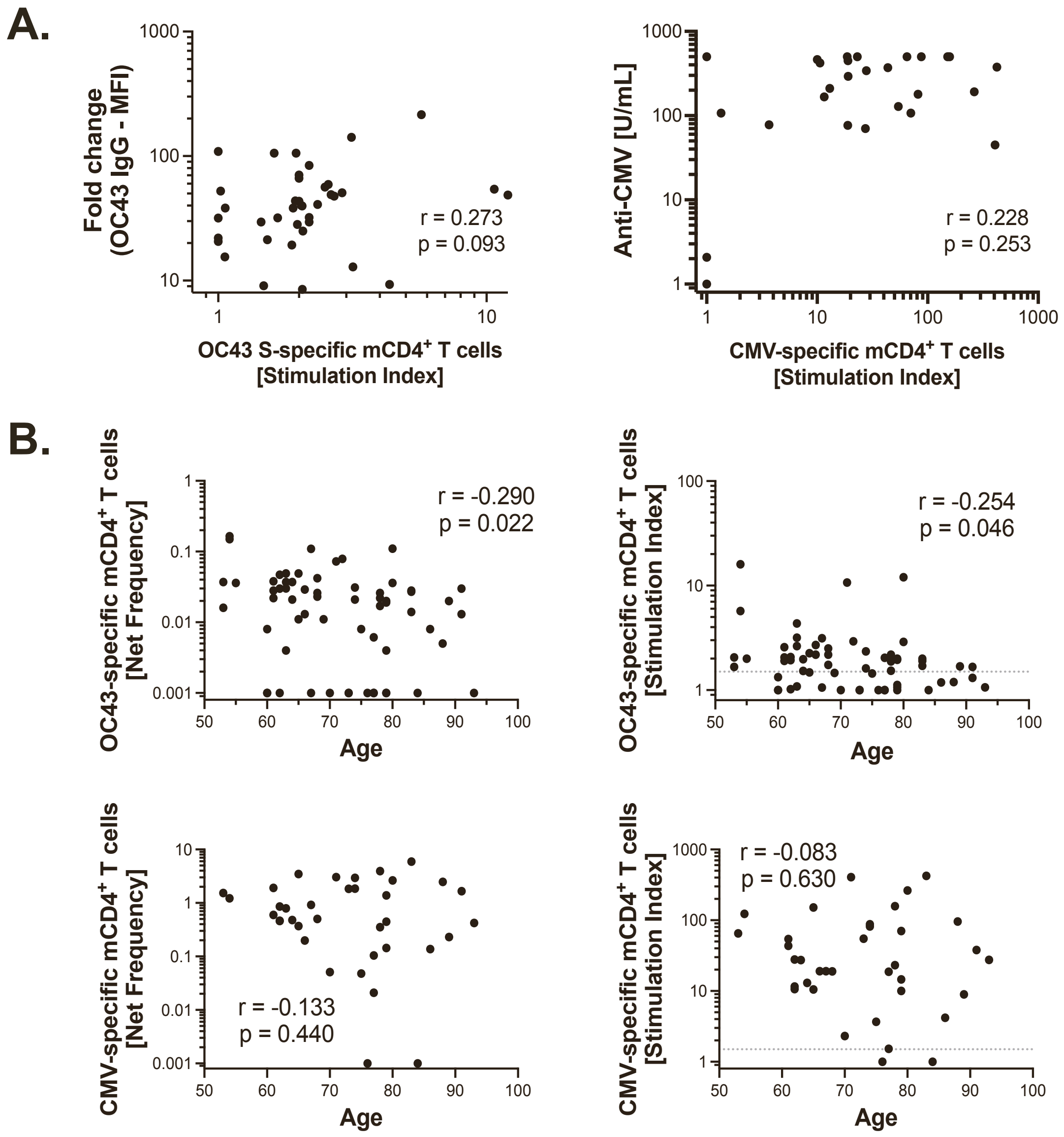

Fig. S3. No correlation between virus-specific mCD4$^+$ T cells and antibody levels, related to Figure 3.

A. Spearman correlation of the stimulation index (SI) of virus-specific mCD4$^+$ T cells and the level of corresponding antibody. Only patients (OC43, n =15; CMV, n = 8) and controls (OC43, n = 24; CMV, n = 19) with both serology and T cell results available were included.

B. Spearman correlation between age (in years) and the frequency and stimulation index of OC43-specific (top) and CMV-specific (bottom) mCD4$^+$ T cells. The grey, dotted lines show the threshold for a positive response. Includes both patients (OC43, n = 34; CMV, n = 18) and controls (OC43, n = 28; CMV, n = 20). Correlation graphs show spearman correlation coefficient (r) and p-value (p). A p-value < 0.05 was considered significant.

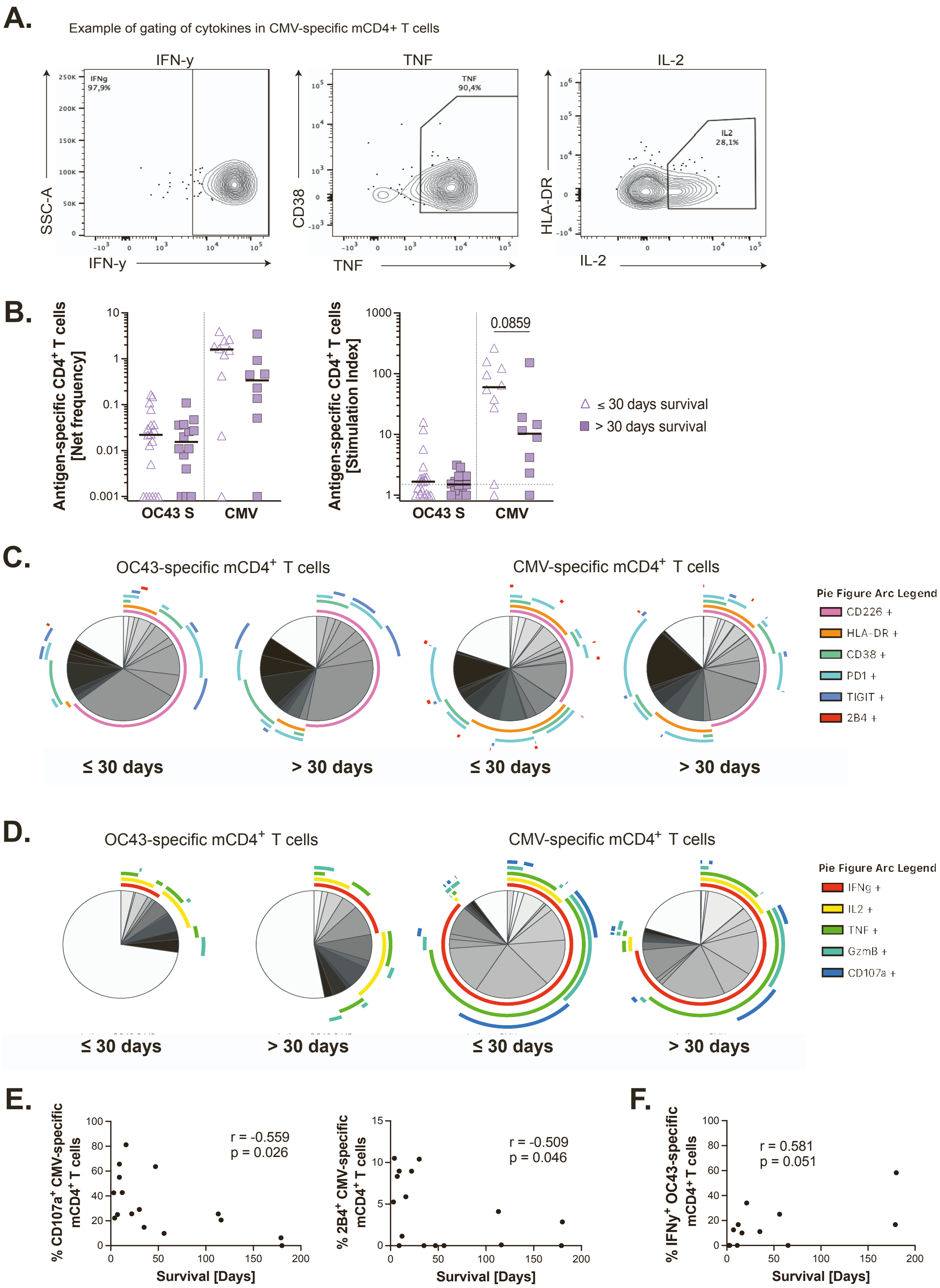
A.
Example of gating of cytokines in CMV-specific mCD4+ T cells
IFN-y
TNF
IL-2
IFNg 97,9%
TNF 90,4%
IL2 28,1%
SSC-A
CD38
HLA-DR
IFN-y
TNF
IL-2
B.
Antigen-specific CD4+ T cells [Net frequency]
Antigen-specific CD4+ T cells [Stimulation Index]
OC43 S
CMV
0.0859
≤ 30 days survival
> 30 days survival
C.
OC43-specific mCD4+ T cells
CMV-specific mCD4+ T cells
Pie Figure Arc Legend
CD226 +
HLA-DR +
CD38 +
PD1 +
TIGIT +
2B4 +
≤ 30 days
> 30 days
≤ 30 days
> 30 days
D.
OC43-specific mCD4+ T cells
CMV-specific mCD4+ T cells
Pie Figure Arc Legend
IFNg +
IL2 +
TNF +
GzmB +
CD107a +
≤ 30 days
> 30 days
≤ 30 days
> 30 days
E.
% CD107a+ CMV-specific mCD4+ T cells
r = -0.559
p = 0.026
Survival [Days]
% 2B4+ CMV-specific mCD4+ T cells
r = -0.509
p = 0.046
Survival [Days]
F.
% IFNy+ OC43-specific mCD4+ T cells
r = 0.581
p = 0.051
Survival [Days]

Fig. S4. CMV-specific mCD4$^+$ T cells maintain their functional capacity in the last month of life, related to Figure 4.

A. Example of gating for IFN-γ, TNF and IL-2 on CMV-specific mCD4$^+$ T cells.

B. Graphs showing the net frequency and stimulation index of antigen-specific mCD4$^+$ T cells in end-of-life patients who survived ≤ 30 days (OC43, n = 19; CMV, n = 10) or > 30 days (OC43, n = 15; CMV, n = 8) after sampling. Mann Whitney U test.

C. Pie chart generated by SPICE showing the phenotypic profile of OC43- and CMV-specific mCD4$^+$ T cells in end-of-life patients who survived ≤ 30 days (OC43, n = 7; CMV; n = 9) or > 30 days (OC43, n = 5; CMV, n = 7) after sampling. The white pie piece represents cells lacking expression of all markers. Only donors with a positive antigen-specific response were included. Permutation test. Data are represented as mean frequency.

D. Pie chart generated by SPICE showing the functional profile of OC43- and CMV-specific mCD4$^+$ T cells in end-of-life patients who survived ≤ 30 days (OC43, n = 7; CMV; n = 9) or > 30 days (OC43, n = 5; CMV, n = 7) after sampling. The white pie piece represents cells lacking expression of all markers. Only donors with positive responses were included. Permutation test. Data are represented as mean frequency.

E. Spearman correlation between the frequency of CMV-specific mCD4$^+$ T cells expressing CD107a (left) or 2B4 (right) and survival time in days post sampling. Only positive donors were included.

F. Spearman correlation between the frequency of IFN-γ$^+$ OC43-specific mCD4$^+$ T cells and survival time in days after sampling. Only positive donors were included. Data represented as the mean proportion.

Correlation graphs show spearman correlation coefficient (r) and p-value (p). *, $p < 0.05$; **, $p < 0.01$; ***, $p < 0.001$; ****, $p < 0.0001$. The median (black line) is shown when applicable.

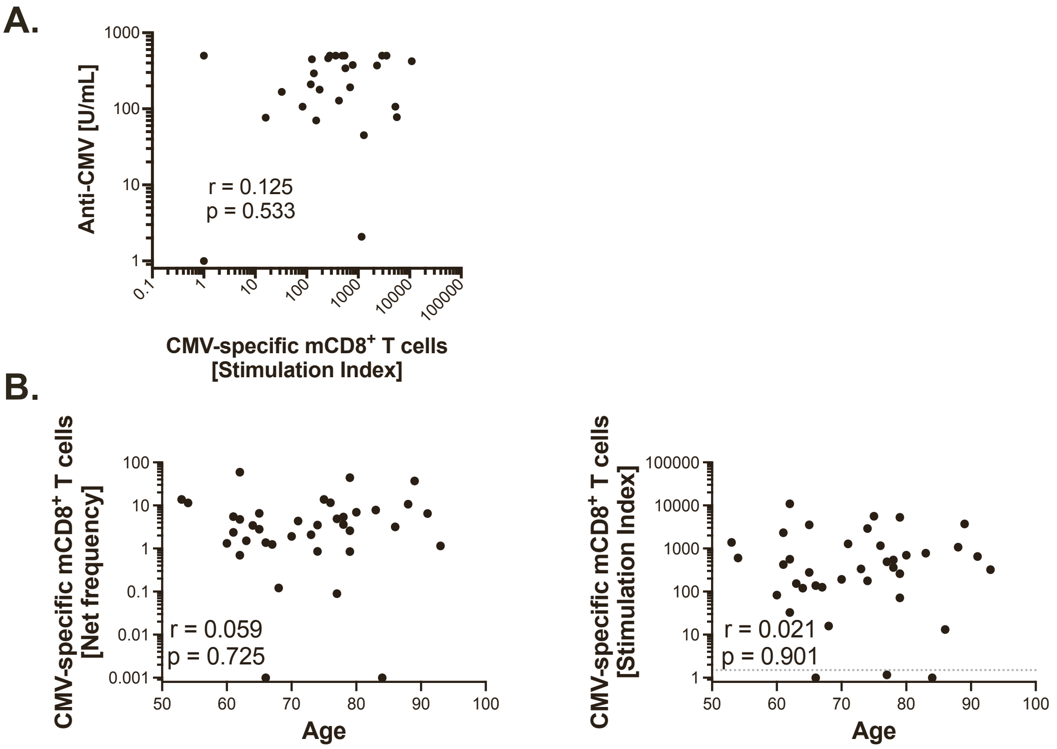

Fig. S5. No correlation between CMV-specific mCD8$^+$T cells and anti-CMV titres, related to Figure 5.

A. Spearman correlation of the stimulation index of CMV-specific mCD8$^+$ T cells and the magnitude of anti-CMV antibodies. Only patients (n=8) and controls (n = 19) with both serology and T cell results available were included.

B. Spearman correlation between age and the frequency (left) and stimulation index (right) of CMV-specific mCD8$^+$ T cells. The grey, dotted line indicates the threshold for a positive T cell response. Includes both patients (n = 18) and controls (n = 20).

Correlation graphs show spearman correlation coefficient (r) and p-value (p). A p-value < 0.05 was considered significant.

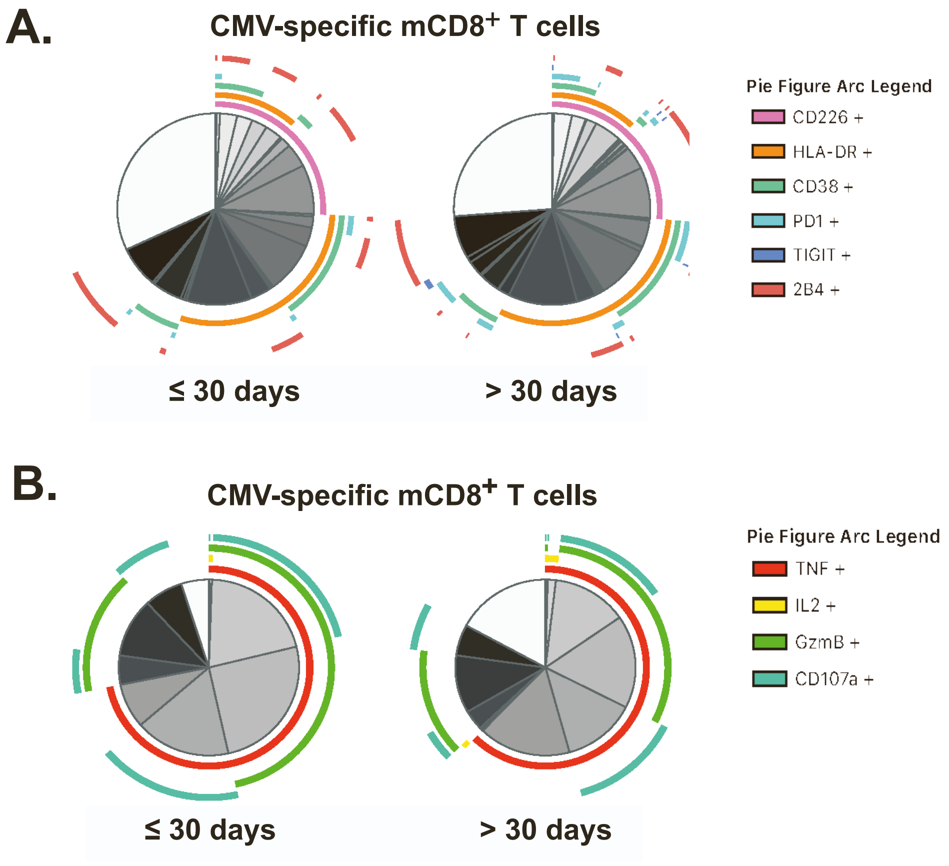

Fig. S6. No change in the phenotypic nor functional profile of CMV-specific mCD8$^+$ T cells in the last month of life, related to Figure 6.

A. Pie chart generated by SPICE showing the phenotypic profile of CMV-specific mCD8$^+$ T cells in end-of-life patients who survived ≤ 30 days (n = 8) or > 30 days (n = 8) after sampling. The white piece represents cells lacking expression of all markers. Only donors with a positive CMV-specific response were included. Permutation test. Data are represented as mean frequency.

B. Pie chart generated by SPICE showing the functional profile of CMV-specific mCD8$^+$ T cells in end-of-life patients who survived ≤ 30 days (n = 8) or > 30 days (n = 8) after sampling. The white piece represents cells lacking expression of all markers. Only donors with a positive CMV-specific response were included. Permutation test. Data are represented as mean frequency.

A p-value < 0.05 was considered significant.
